# Unified artificial intelligence framework for modeling pollution dynamics and sustainable remediation in environmental chemistry

**DOI:** 10.1038/s41598-025-20083-w

**Published:** 2025-10-16

**Authors:** Mohammad Fazle Rabbi

**Affiliations:** Coordination and Research Centre for Social Sciences, Faculty of Economics and Business, University of Debrecen, Böszörményi út 138, Debrecen, 4032 Hungary

**Keywords:** Artificial intelligence, Environmental chemistry, Pollution modeling, Physics informed neural networks, Sustainable remediation, Engineering, Environmental sciences, Mathematics and computing

## Abstract

Environmental pollution involves complex nonlinear interactions, chemical variability, and physical constraints that challenge traditional models. A unified artificial intelligence framework integrating Graph Neural Networks, Generative Adversarial Networks, Reinforcement Learning, Green Chemistry optimization, and Physics Informed Neural Networks with embedded Darcy’s law and a hybrid AI physics model. This framework simulates contaminant transport, generates climate scenarios, and optimizes sustainable remediation strategies across four calibrated environmental scenarios. Validation employed four synthetic environmental scenarios with parameters calibrated from documented PFAS contamination studies, representing controlled algorithm development prior to field deployment (noise sigma 1.5 to 4.0 mg per liter; seasonal amplitude 0.1 to 0.3; trend 0 to 0.1 mg per liter per day). The hybrid AI physics model achieved 89% predictive accuracy on synthetic validation datasets with literature-calibrated parameters, outperforming traditional (65%), pure AI (78%), and physics-only (72%) approaches under controlled synthetic conditions. Graph Neural Networks captured complex spatiotemporal patterns (R² > 0.89), while Reinforcement Learning optimization improved simulated treatment efficiency from 62.3% to 89.7% in synthetic remediation scenarios. Green Chemistry optimization modeling suggested supercritical carbon dioxide and ionic liquids as potentially efficient solvents, with model-predicted efficiencies of 88% to 92% and relative toxicity scores between 1.8 and 2.1 units (where lower scores indicate reduced environmental impact). Physics Informed Neural Networks reduced physics loss from approximately 1.2 to 0.03 ± 0.005, achieving convergence at a total loss of 0.08 ± 0.01 over 50 training epochs on synthetic datasets. The framework demonstrated computational scalability from 80 to 5000 synthetic records and estimated deployment feasibility within a 22-month timeline under optimal conditions, contingent upon successful field validation. SHAP and LIME analyses indicated natural attenuation, particularly the decay process, as the most influential model feature, contributing a mean SHAP value of 0.34 ± 0.08, consistent with expected physical processes. This framework contributes to accurate, interpretable, and sustainable pollution modeling.

**Supplementary Information:**

The online version contains supplementary material available at 10.1038/s41598-025-20083-w.

## Introduction

Environmental systems present complex chemical interactions and variability that challenge traditional modeling methods^1^. Despite the advancements in computational techniques, these methods frequently rely on simplified assumptions, encounter challenges with sparse data, and incur substantial computational costs for long-term, high-resolution simulations^2,3^. Emerging artificial intelligence approaches can overcome these limitations by blending data-driven inference with physical and sustainability constraints^4^. However, this discipline has undergone a fundamental metamorphosis, propelled by the synergistic forces of advanced computational modeling, the interdisciplinary insights gleaned from systems science, and an urgent global push towards sustainability driven innovation^5^. The challenges are formidable as pollution dynamics are growing in complexity. This is fueled by intensifying human activities, insidious climate feedback loops, and the relentless emergence of novel contaminants in our ecosystems. Confronted with such multifaceted problems, conventional modeling techniques frequently prove deficient in capturing the nonlinear, interconnected, and multi-scalar behavior inherent to environmental systems^6^. The necessity for advanced simulation tools is evident in various contexts, ranging from the complex spread of groundwater contamination in subterranean environments to the substantial and often imperceptible atmospheric dispersion of industrial emissions. This necessity further extends to the molecular design of biodegradable materials and the development of advanced remediation technologies, underscoring the paramount demand for sophisticated, adaptable, and predictive simulation tools in addressing these challenges^7^. Conventional approaches, constrained by simplified assumptions and linear approximations, are increasingly inadequate in addressing the dynamic realities of our polluted world. These approaches face challenges in accurately forecasting tipping points, comprehending emergent phenomena, or predicting the long-term consequences of complex pollutant mixtures^8,9^.

Robust predictive and prescriptive capabilities are critically needed in environmental chemistry. Nevertheless, a significant limitation exists in current technological tools, particularly regarding the improved understanding, simulation, and mitigation of complex pollution dynamics^10–12^. While computational modeling has indeed advanced, its traditional implementations frequently lack the necessary agility and foresight to effectively contend with the dynamic, interconnected, and highly heterogeneous nature of environmental systems across their vast spatiotemporal scales. Addressing the imperative for high-resolution, long-duration simulations and the comprehensive exploration of diverse remediation scenarios is hindered by current models. These tools are often criticized for their oversimplification, challenges with sparse or incomplete data sets, and a significant computational resource demands. Such limitations have a considerable impact on the accuracy of forecasts of pollution progression, the assessment of the environmental efficacy of proposed remediation strategies in computational models prior to the allocation of substantial resources to physical deployment, and the proactive design of materials and chemical processes with an inherently minimal environmental footprint^13–15^. Ineffective simulation of these intricate environmental interactions prevents sound decision making. This leads invariably to suboptimal pollution control measures and fosters a reactive, rather than an accurately preventive, approach to safeguarding the shared environment. Consequently, there is an urgent and undeniable demand for Artificial Intelligence AI driven approaches, particularly those harnessing the power of machine learning and hybrid computational models. Advanced AI techniques promise to resolve these existing challenges. They enable the extraction of valuable insights from data, allow for efficient creation of various scenarios to assess risks, and support the improvement of environmental interventions^16,17^. This goes beyond the reach of conventional scientific approaches. The adoption of artificial intelligence provides a means to synthesize empirical observations, theoretical frameworks, and practical environmental solutions. This synthesis is critically necessary for effectively navigating the intricate chemical challenges facing the planet.

Despite these advances, several critical challenges persist. Many AI applications in environmental chemistry remain narrowly focused on predictive accuracy, often at the expense of physical interpretability or sustainability alignment^18^. Purely data-driven models may perform well in controlled settings but tend to generalize poorly to real-world systems with limited or imbalanced data^19^. Furthermore, deep embedding of domain specific scientific knowledge into machine learning architectures, especially for materials science or chemistry applications, is possible via a synergistic combination of advanced AI theories^20^. One compelling approach involves Graph Neural Networks GNNs to model molecular structures or reaction networks, where atoms and bonds form nodes and edges, allowing the inherent connectivity and chemical rules to be represented^21^. Simultaneously, Generative Adversarial Networks GANs could be employed to create novel molecular designs or synthetic pathways that adhere to desired properties, with the discriminator guided by scientific principles to assess the “realism” and adherence to chemical validity^22^. Furthermore, Reinforcement Learning RL agents could explore vast chemical spaces, learning optimal synthesis routes or material compositions by receiving rewards based on their adherence to physical laws or achieving specific performance targets^23^. Green Chemistry Principles can exert a substantial influence on this learning process. Metrics like atom economy or toxicity prediction can be embedded directly into the reward function, thereby steering the discovery mechanism toward environmentally benign solutions^24^. In addition, Physics Informed Neural Networks (PINNs) enable the direct incorporation of fundamental physical and chemical laws, including quantum mechanical constraints or reaction kinetics, into the neural network’s loss function. This integration ensures that all generated or predicted outcomes are not merely statistically plausible but also possess scientific coherence and physically realistic^25^. Consequently, the strategic integration of such advanced AI techniques significantly enhances the capability of environmental AI systems to deliver reliable, generalizable, and environmentally meaningful solutions to pressing pollution and remediation challenges.

In the current landscape, artificial intelligence applications in environmental chemistry often remain highly domain specific and rarely integrate fundamental physical laws or comprehensive sustainability objectives, highlighting a significant opportunity for advancement in AI methodologies^26–28^. Existing frameworks seldom combine diverse AI approaches with green chemistry and physics-based constraints into a unified system capable of robustly simulating pollution dynamics and supporting sustainable remediation decisions, especially under conditions of uncertainty and limited data availability. Furthermore, limited work has been dedicated to developing simulation-based methodologies that concurrently predict pollutant behavior and guide decision-making for environmentally sustainable interventions while addressing the inherent challenges of sparse field data through systematic synthetic-to-real validation frameworks.

While individual AI models have made notable progress in environmental applications, a comprehensive framework that integrates a broad spectrum of AI techniques with explicit physical and green chemistry constraints remains undeveloped. To address this gap systematically, this study employs synthetic data generation with literature-calibrated parameters as a methodological foundation for controlled algorithm development. This approach enables rigorous comparison of AI methodologies under known ground truth conditions while establishing the framework for subsequent field validation. Consequently, this study formulates the following research questions: (RQ1) How can AI models simulate environmental chemistry processes using synthetic data generation that maintains environmental realism? (RQ2) What are the comparative strengths of diverse AI frameworks in environmental systems through synthetic validation and literature-calibrated scenarios? (RQ3) In what ways can data-driven learning be meaningfully constrained by physical laws and green chemistry principles to enhance model robustness and ecological validity?.

To address these questions, the primary objective is to develop, rigorously compare, and apply a suite of AI-driven simulation models encompassing graph-based learning for pollutant interactions, generative adversarial networks for environmental scenario synthesis, reinforcement learning for remediation optimization, green chemistry-informed multi-objective optimization for solvent selection, and physics-informed neural networks embedding transport equations. The framework employs literature-calibrated synthetic data generation to enable controlled algorithm development while maintaining environmental realism, establishing a methodological foundation for subsequent real-world validation studies. Hybrid AI physics models are emphasized due to their ability to synergistically combine data driven predictions with domain specific constraints, such as Darcy’s law for porous media flow^29^ and green chemistry principles^30^. Through systematic conceptual formulation, scenario-based simulation, and extensive validation, this work contributes a robust and integrated computational framework aimed at advancing sustainability-focused environmental chemistry.

## Literature review

### Applications of artificial intelligence in environmental chemistry

Artificial intelligence techniques have transformed environmental chemistry by enabling the capture of complex nonlinear interactions, multivariate dependencies, and spatiotemporal dynamics that exceed the capacity of conventional mechanistic models. Recent work demonstrates machine learning and neural network algorithms forecasting contaminant dispersion in soil and aquatic systems with improved accuracy over empirical methods^31,32^. Generative adversarial networks have been implemented to synthesize realistic pollution scenarios under limited observational data, aiding in climate-induced event prediction and stress testing^33,34^. Reinforcement learning agents have optimized dynamic remediation schedules in water treatment applications, achieving notable gains in resource efficiency and contaminant removal rates^35,36^. Green-chemistry informed frameworks apply AI-driven multi-objective optimization to chemical synthesis, balancing reaction yield with reduced environmental impact through tailored solvent selection and energy minimization^37,38^.

Achieving integrated environmental assessments and robust decision support critically requires interoperable artificial intelligence solutions. However, many current AI deployments remain confined to specific domains, thus do not meet this fundamental requirement^12,38,39^. The omission of physical constraints and sustainability metrics in purely data-driven models undermines interpretability and generalization to novel conditions, particularly when field data are sparse or imbalanced^40^. A substantial analytical challenge in the rapidly evolving field of artificial intelligence is the limited availability of comprehensive comparative studies that encompass its numerous paradigms. This phenomenon significantly hinders a comprehensive understanding of their respective strengths, weaknesses, and optimal applications. Parallel to these trends, hybrid approaches are now beginning to constitute a significant and promising new frontier^41,42^. These innovative methods ingeniously embed specific, often critical, domain knowledge such as fundamental conservation laws in physics or precise ecotoxicity criteria relevant to environmental science^43^. This integration of specialized expertise promises to yield more robust, interpretable, and ultimately more effective AI solutions. Continued development of unified frameworks that merge empirical learning with physical and green-chemistry principles is essential to extend AI’s applicability to real-world environmental challenges.

### Theoretical foundations of AI approaches

To address the identified shortcomings of purely data-driven and isolated AI implementations, the current study integrates six foundational artificial intelligence approaches with proven relevance to environmental chemistry. Specifically, these approaches integrate: graph neural networks for pollutant interaction modeling; generative adversarial networks for simulating future pollution scenarios; reinforcement learning for optimizing intervention strategies; green chemistry based multi-objective optimization for sustainable material and process design; physics informed neural networks for embedding environmental laws into model training; and hybrid AI physics frameworks, which combine data driven learning with physics based constraints.

Table 1 summarizes these frameworks by detailing each theory’s originator, core mechanisms, a representative environmental application, primary methodological limitations, and specific contributions to environmental chemistry challenges. This synthesis establishes the conceptual foundation for the simulation methodologies developed in this work and underpins a comparative analysis of how individual AI paradigms can be strategically combined to enhance pollutant modeling and remediation decision making in complex environmental systems.

**Table 1:** Core AI theories and their applications in environmental chemistry.

Theory	Originator	Key Concepts	Representative Application	Primary Limitations	Relevance to AI & Environmental Chemistry
1. Graph Neural Networks (GNNs)	Scarselli et al. (2009)^44^	– Node feature aggregation – Graph convolution	Soil-water contaminant dispersion modeling	– Scalability issues – Dynamic graph handling	Captures relational pollutant pathways and identifies critical remediation hubs
2. Generative Adversarial Networks (GANs)	Goodfellow et al. (2014)^45^	– Adversarial training – Implicit density estimation	Synthesis of climate-driven pollution scenarios	– Mode collapse – Extreme-event instability	Enables realistic scenario generation under scarce observational data
3. Reinforcement Learning (RL)	Sutton and Barto (1998)^46^	– Markov decision processes – Reward maximization	Dynamic remediation scheduling in water treatment	– Sample inefficiency – Continuous action instability	Optimizes adaptive intervention strategies to improve treatment efficiency
4. Green Chemistry Optimization	Anastas and Warner (2000)^30^	– Multi-objective loss (waste, energy, toxicity) – Pareto front	AI-driven solvent selection for sustainable synthesis	– Sparse eco-friendly data – Workflow integration	Guides sustainable materials and process design by balancing eco-metrics
5. Physics-Informed Neural Networks (PINNs)	Raissi et al. (2019)^47^	– Differential operator embedding – Loss regularization	CO₂ transport modeling in porous media	– High computational cost – Requires domain expertise	Embeds conservation laws into learning for physically coherent predictions
6. Hybrid AI-Physics Frameworks	Huynh et al. (2025)^39^	– Weighted AI-physics loss – Joint optimization	Integrated contaminant migration and remediation modeling	– Complex hyperparameter tuning – Parameter dependency	Combines data-driven and physics constraints to enhance accuracy and interpretability

This table provides a comparative synthesis of each AI theory’s conceptual origin, core mechanisms, representative environmental applications, primary methodological limitations, and distinctive contributions to pollutant modeling and remediation decision support.

## Methodology

Building upon the theoretical foundations outlined in the literature review, this study employs a suite of six advanced artificial intelligence modeling frameworks, each tailored to simulate a distinct dimension of pollution dynamics and environmental remediation. The models selected for this study, including Graph Neural Networks (GNN), Generative Adversarial Networks (GAN), Reinforcement Learning (RL), Green Chemistry multi objective optimization, Physics Informed Neural Networks (PINN), and a hybrid AI physics framework, were chosen due to their proven applicability in environmental contexts and their complementary ability to capture both data driven and physics-based processes.

Each framework is presented with its mathematical formalism and implementation details to ensure full methodological transparency and reproducibility.

### Graph neural network framework for pollutant interaction modeling

The Graph Neural Networks (GNNs) approach models, which are particularly effective for representing structured relationships within environmental systems, such as the spatial and temporal interactions between pollutants and soil matrices. GNNs iteratively update node features by aggregating information from neighboring nodes, enabling predictive modeling in complex networks like soil-pollutant or river-contaminant systems.

1 $$\begin{aligned}{h}_{v}^{(l+1)}=\sigma\:\left(\sum\limits_{u\in\:\mathcal{N}\left(v\right)}\:\:{W}^{\left(l\right)}{h}_{u}^{\left(l\right)}\right)\end{aligned}$$

The GNN implementation models pollutant interactions by updating node features $\:{h}_{v}$ via aggregation of neighbor data, as specified in Eq. (1). Weight matrices $\:{W}^{\left(l\right)}$ were initialized using Xavier initialization to promote stable convergence. This architecture is implemented to predict phenomena such as PFAS adsorption kinetics, with the network topology and simulation setups precisely reflecting the environmental scenarios.

### Generative adversarial networks (GANs) emulation of climate scenarios

Generative Adversarial Networks (GANs) are employed to simulate future environmental scenarios, including long-term climate patterns and rare pollution events. Within GANs, a generator neural network creates synthetic data while a discriminator neural network evaluates its authenticity through adversarial training, ultimately allowing for the synthesis of realistic environmental data distributions. This approach is particularly useful in domains where observational data is sparse or biased.

2 $$\:\underset{G}{\text{m}\text{i}\text{n}}\:\underset{D}{\text{m}\text{a}\text{x}}\:V(D,G)={\mathbb{E}}_{x\sim\:{p}_{\text{data}}}[\text{l}\text{o}\text{g}D(x\left)\right]+{\mathbb{E}}_{z\sim\:{p}_{z}}[\text{l}\text{o}\text{g}(1-D\left(G\right(z\left)\right)\left)\right]$$

Trains a generator $\:G\:$ to produce realistic climate data (e.g., 100-year ice-melt trajectories) 25x faster than physics models. Fails to capture extreme events due to training data biases.

### Reinforcement learning (RL) optimization for remediation scheduling

The third model type is Reinforcement Learning (RL), which is leveraged to optimize dynamic environmental interventions such as remediation scheduling or resource allocation. RL operates through iterative learning via trial-and-error, guided by reward functions that encode environmental performance criteria. This framework is well-suited to sequential decision-making problems encountered in pollution control.

3 $$\begin{aligned}{V}^{\text{*}}\left(s\right)=\underset{a}{\text{m}\text{a}\text{x}}\:\left[R(s,a)+\gamma\:\sum\limits_{{s}^{{\prime\:}}}\:\:P\left({s}^{{\prime\:}}\right|s,a\left){V}^{\text{*}}\right({s}^{{\prime\:}})\right]\end{aligned}$$

Optimizes remediation strategies (e.g., dynamic allocation of groundwater treatment resources) with 30–50% cost savings. Sample inefficiency limits scalability to large ecosystems.

### Green Chemistry-Informed multi-objective optimization

Complementing these data-centric methods, the Green Chemistry Principles are integrated into an AI-assisted optimization framework. This framework guides the selection of materials and processes toward minimal environmental impact by evaluating tradeoffs between metrics such as waste, toxicity, and energy use^48^thereby aligning computational modeling with sustainable chemistry design. The optimization problem is formally defined as:

4 $$\:\underset{x}{\text{m}\text{i}\text{n}}\:\left[\alpha\:\cdot\:\text{Waste}\left(x\right)+\beta\:\cdot\:\text{Energy}\left(x\right)+\gamma\:\cdot\:\text{Toxicity}\left(x\right)\right]$$

where $\:x$ represents the material or process design variables, and $\:\alpha\:$, $\:\beta\:$, $\:\gamma\:$ are user defined weighting coefficients for the respective environmental impact metrics.

### Physics-informed neural network modeling of CO₂ transport

Physics Informed Neural Networks (PINNs) provide a robust approach by embedding fundamental transport equations directly into their training objectives. This ensures that model predictions adhere to both observed data and known environmental physics. In this methodology, the loss function combines a data fidelity term and a physics-based term:

5 $$\:\mathcal{L}={\lambda\:}_{\text{data}}\Vert\:{u}_{\text{NN}}-{u}_{\text{obs}}{\Vert\:}^{2}+{\lambda\:}_{\text{physics}}\Vert\:\mathcal{F}\left({u}_{\text{NN}}\right){\Vert\:}^{2}$$

Here, $\:{u}_{\text{NN}}$ represents the neural network’s prediction, $\:{u}_{\text{obs}}$ are the observed data points, and $\:F\left(\cdot\:\right)$ denotes operators such as advection diffusion or Darcy’s law for porous media flow. The coefficients $\:{{\uplambda\:}}_{data}$ and $\:{{\uplambda\:}}_{physics}$ balance the weight of data fit against physical consistency. By penalizing deviations from these governing equations alongside measurement errors, the network avoids nonphysical artifacts and enforces conservation principles, even under conditions of sparse field data. This dual objective formulation effectively regularizes the model, enhancing its capacity to generalize to unseen conditions and improve predictive reliability. Optimal performance requires careful calibration of $\:{{\uplambda\:}}_{data}$ and $\:{{\uplambda\:}}_{physics}$​, typically guided by methods such as cross validation or Bayesian optimization.

### Hybrid AI-physics integration for contaminant migration

Developing a hybrid AI physics modeling framework involves the fusion of Graph Neural Network (GNN) based learning and physics-based loss functions. This approach specifically integrates fundamental environmental laws, exemplified by the inclusion of Darcy’s law for fluid flow in porous media. This hybrid methodology leverages both data driven insights and domain specific constraints to improve predictive accuracy in complex environmental scenarios, such as contaminant migration in thawing permafrost.

The hybrid loss function, which combines a graph neural network (GNN) term $\:{\mathcal{L}}_{\text{GNN}}$ with Darcy’s law $\:{\mathcal{L}}_{Darcy}$, is defined as:

6 $$\:{\mathcal{L}}_{\text{hybrid}}={\lambda\:}_{\text{AI}}\cdot\:{\mathcal{L}}_{\text{GNN}}+{\lambda\:}_{\text{physics}}\cdot\:{\mathcal{L}}_{\text{Darcy}}$$

Here $\:{\lambda\:}_{AI}$ and $\:{\lambda\:}_{physics}$ are weighting parameters that balance the contributions of the data driven GNN component and the physics-based constraint. This formulation enables the model to effectively simulate contaminant migration in porous media (e.g., PFAS in thawing permafrost) by enforcing both data driven predictions and adherence to physical flow constraints.

Together, these six modeling strategies form the methodological backbone of this study. Each is introduced below with its mathematical formulation and contextual justification, comprising a comprehensive toolkit for simulating environmental phenomena via AI enhanced sustainability science.

### Synthetic data generation protocol

Environmental scenarios were generated using a comprehensive simulation framework that systematically combines multiple environmental processes to create realistic pollutant concentration trajectories while addressing methodological transparency concerns raised by peer reviewers. The synthetic data generation protocol integrates exponential decay representing natural attenuation processes, seasonal oscillations reflecting climate-driven variations, linear trends accounting for long-term pollution accumulation or depletion, and stochastic noise components representing measurement uncertainty and environmental variability inherent in real monitoring systems. This synthetic framework provides controlled conditions for algorithm development and comparison while maintaining environmental realism through parameters calibrated from documented PFAS contamination studies. The base concentration of 50.0 mg/L and decay rate of 0.05 day⁻¹ directly correspond to PFAS persistence characteristics reported in military installations and industrial facilities, establishing a foundation for the literature-parameterized validation analysis presented in Sect. 4.7, Fig. 7. However, this study employs synthetic data generation as a preliminary validation step essential for controlled algorithm development before field deployment. While synthetic scenarios enable systematic performance comparison under known ground truth conditions, they represent idealized environmental conditions with controlled parameter ranges. The literature-calibrated approach provides environmental realism within the constraints of simplified mathematical models, establishing methodological foundations for subsequent real-world validation studies.

This mathematical framework enables systematic exploration of environmental scenarios while maintaining physical realism through calibrated parameters derived from environmental monitoring literature. The fundamental mathematical formulation governing the synthetic concentration dynamics follows a superposition principle where individual environmental processes are additively combined to capture the multifaceted nature of real environmental systems. The temporal evolution of pollutant concentration $\:C\left(t\right)$ at time t is expressed as:

7 $$\:C\left(t\right)={C}_{0}\cdot\:\text{e}\text{x}\text{p}(-k\cdot\:t)+{A}_{seasonal}\cdot\:\text{s}\text{i}\text{n}\left(\frac{2\pi\:t}{365.25}\right)+\beta\:\cdot\:t+N(0,{\sigma\:}_{noise})$$

The exponential decay component represents the dominant natural attenuation mechanism characteristic of biodegradable contaminants in environmental systems, providing the foundation for realistic concentration trajectories. The base concentration parameter C₀ was calibrated to 50.0 mg/L, representing typical PFAS contamination levels encountered in environmental monitoring studies and consistent with reported ranges in peer-reviewed literature for persistent organic pollutants in groundwater and surface water systems.

The decay rate constant $\:k$ was set to 0.05 day⁻¹, derived from biodegradation kinetics reported in environmental chemistry literature for similar persistent organic pollutants under aerobic conditions. This parameter reflects the natural attenuation capacity of environmental systems through microbial degradation, photolysis, and chemical transformation processes that collectively reduce contaminant concentrations over time.

The seasonal amplitude parameter $\:{A}_{seasonal}$ varies between 0.1 and 0.3 times the base concentration, reflecting the magnitude of seasonal transport variations observed in natural systems due to temperature-dependent processes, precipitation patterns, and biological activity cycles. The linear trend coefficient β accounts for systematic long-term changes in contamination levels, with values ranging from 0.0 to 0.1 mg/L per day depending on the specific environmental scenario being modeled. This parameter represents ongoing contamination sources, remediation effectiveness, or climate-driven changes in contaminant mobility that produce systematic increases or decreases in environmental concentrations over extended monitoring periods.

The stochastic noise component $\:N(0,{\sigma\:}_{noise})$ follows a normal distribution with zero mean and standard deviation $\:{\sigma\:}_{noise}$ ranging from 1.5 to 4.0 mg/L, representing the combined effects of measurement uncertainty, small-scale environmental variability, and unmodeled processes that contribute to observed concentration fluctuations in real monitoring data. This component accounts for analytical precision limitations, spatial heterogeneity effects, and short-term environmental perturbations that are not captured by the deterministic model components.

#### Scenario-Specific parameter configuration and environmental context

To rigorously evaluate model performance across diverse contamination contexts, four environmental scenarios were systematically implemented. Each configuration was carefully designed to represent characteristic contamination dynamics within real-world environmental systems. This explicit parameter documentation ensures full methodological transparency and reproducibility.

The base scenario serves as the reference, employing a moderate noise standard deviation (σ) of 2.0 mg/L. Seasonal fluctuations are minimal, set with an amplitude coefficient of 0.1 times the base concentration, reflecting stable conditions with limited climate-driven variability. The trend coefficient (β) is fixed at zero, signifying the absence of systematic long-term contamination changes. The scenario thus typifies well-characterized contamination sites with steady-state operations and consistent monitoring, typical of established contamination plumes under natural attenuation conditions, similar to legacy industrial sites with well-documented baseline conditions.

Expanding in complexity, the high variability scenario is configured to simulate climate-stressed systems subjected to frequent environmental perturbations. Here, a higher noise level (σ = 4.0 mg/L), seasonal amplitude (0.2), and a moderate positive trend coefficient (β = 0.05 mg/L·day⁻¹) are used. Such settings mirror regions affected by extreme weather, stress responses, or accelerated pollutant release, representative of climate-stressed systems such as those observed in Arctic regions with permafrost thaw or coastal areas affected by extreme weather events.

Turning towards systems dominated by cyclic patterns, the seasonal dominant scenario emphasizes periodicity with the maximum seasonal amplitude (0.3), reduced noise (σ = 1.5 mg/L), and a minimal trend (β = 0.02 mg/L·day⁻¹). This setup is characteristic of agricultural watersheds where fertilizer application cycles and crop management practices drive pronounced temporal concentration patterns.

Finally, the trend dominant scenario represents environments experiencing systematic contaminant accumulation. Elevating the noise (σ = 2.5 mg/L) but minimizing seasonal amplitude (0.05) and intensifying the trend (β = 0.1 mg/L·day⁻¹), it is tailored to legacy or ongoing industrial discharge contexts, where contaminant concentrations rise persistently due to continuous inputs or evolving conditions, reflecting ongoing contamination sources or legacy sites with increasing contaminant mobilization, as documented in several military and industrial facilities.

A consolidated summary of all scenario defining parameters, including noise, seasonal amplitude, trend, base concentration, decay rate, time span, and contextual application, is provided in Table 2. These values are the direct simulation inputs (as per Eq. 7), thereby ensuring full reproducibility and transparency.

**Table 2:** Environmental scenario parameter configuration and contextual applications.

Scenario	Noise σ (mg/L)	Seasonal Amplitude	Trend Coefficient (mg/L·day⁻¹)	Base Conc. (mg/L)	Decay Rate (day⁻¹)	Time Span (days)	Environmental Context
Base	2.0	0.1	0.00	50.0	0.05	200	- Stable contamination site - Consistent monitoring - Steady-state industrial facility
High Variability	4.0	0.2	0.05	50.0	0.05	200	- Climate-stressed system - Extreme weather impacts - Accelerated pollutant release (e.g., thaw, erosion)
Seasonal Dominant	1.5	0.3	0.02	50.0	0.05	200	- Agricultural runoff system - Pronounced seasonal cycles - Fertilizer & crop-driven transport
Trend Dominant	2.5	0.05	0.10	50.0	0.05	200	- Industrial accumulation - Ongoing discharges - Increasing contaminant levels

All parameter values correspond to the synthetic simulation definitions described in Eq. 7 (Sect. 3.7), ensuring transparent scenario configuration.

By presenting this systematic parameterization in Table 2, direct quantitative comparisons across scenarios are enabled, while providing the methodological rigor essential for reproducible environmental modeling studies. In subsequent analysis, references to scenario performance and behavior always anchor back to these explicit definitions, reinforcing transparency throughout the work.

### Comprehensive model validation framework

Model performance was evaluated using a comprehensive suite of statistical metrics designed to assess different aspects of predictive accuracy and model reliability while addressing reviewer concerns regarding validation rigor. The validation protocol employed Root Mean Square Error (RMSE) to quantify overall prediction accuracy with particular sensitivity to large deviations, Mean Absolute Error (MAE) providing robust assessment less sensitive to outliers, and the coefficient of determination (R²) measuring the proportion of variance explained by the model predictions.

Additional validation metrics included Willmott’s Index of Agreement (d), which accounts for both systematic bias and random error while providing a standardized measure bounded between 0 and 1. This metric was calculated as:

8 $$\begin{aligned}d=1-\frac{{\sum}_{i=1}^{n}\:({P}_{i}-{O}_{i}{)}^{2}}{{\sum}_{i=1}^{n}\:\left(\right|{P}_{i}-\overset{\lower0.5em\hbox{$\smash{\scriptscriptstyle\leftharpoonup}$}} {O}|+|{O}_{i}-\overset{\lower0.5em\hbox{$\smash{\scriptscriptstyle\leftharpoonup}$}} {O}|{)}^{2}}\end{aligned}$$

where $$\:{P}_{i}$$ represents predicted values, $$\:{O}_{i}$$ denotes observed values, and $$\overset{\lower0.5em\hbox{$\smash{\scriptscriptstyle\leftharpoonup}$}} {O}$$ is the mean of observed values. Values approaching unity indicate superior model agreement with observational data, while values below 0.5 suggest poor model performance requiring additional calibration or structural modifications.

The Normalized Root Mean Square Error (NRMSE) was computed by dividing RMSE by the range of observed values, providing scale-independent error assessment suitable for comparing performance across scenarios with different concentration ranges. To further enhance statistical validation, bias assessment was conducted by calculating the mean difference between predicted and observed values. This enabled the identification of systematic over or under estimation tendencies, potentially indicating parameter miscalibration or structural model deficiencies.

Cross-validation employed five-fold stratified sampling to ensure robust performance estimates across all environmental scenarios while maintaining representative distributions in training and validation subsets. This approach prevents overfitting to specific environmental conditions while ensuring that model performance estimates reflect generalization capability across diverse contamination scenarios representative of real-world applications. This comprehensive synthetic validation framework provides the foundation for subsequent real-world validation using actual PFAS contamination data, ensuring that synthetic training conditions appropriately prepare AI models for practical environmental applications while maintaining known ground truth for controlled comparison of modeling approaches.

#### Statistical validation and uncertainty assessment

The comprehensive validation framework demonstrates robust statistical properties that confirm the reliability and representativeness of the synthetic data generation approach across all environmental scenarios. Monte Carlo simulation with 1000 iterations assessed parameter uncertainty distributions, revealing decay rate coefficients of 0.05 ± 0.008 day⁻¹ within 95% confidence intervals, base concentration values of 50.0 ± 2.1 mg/L, and seasonal amplitude variations of ± 15% depending on scenario-specific configurations.

Model predictions maintain coefficient of determination values exceeding 0.85 across all uncertainty ranges, confirming robust predictive performance despite parameter variability inherent in environmental systems. These comprehensive statistical metrics confirm that synthetic data generation maintains statistical fidelity to real environmental systems while enabling controlled comparison of artificial intelligence methodologies under known ground truth conditions, which represents an essential validation step before deployment in real-world environmental monitoring applications.

### Computational implementation and data analysis framework

The computational workflows were implemented using Python 3.12.4, leveraging enhanced interactive interpreter capabilities and Just-in-Time (JIT) compilation features for computationally intensive environmental simulations. The framework utilizes NumPy 1.26.4 for core numerical computing with enhanced parallel processing support, pandas 2.2.2 for time-series environmental data manipulation, and scikit-learn 1.5.1 for machine learning pipeline implementation with expanded model evaluation utilities including environmental-specific validation metrics.

Advanced visualization capabilities employ matplotlib 3.8.4 for publication-quality figures with enhanced plotting performance, while seaborn 0.13.2 provides statistical plotting and data exploration through improved categorical plotting functions suited for environmental scenario comparisons. Network analysis of pollutant transport pathways utilizes NetworkX 3.3 with optimized graph algorithms for modeling contaminant connectivity patterns. Scientific computing operations leverage SciPy 1.13.1, providing improved performance for differential equation solving and enhanced numerical stability for physics-informed neural network implementations.

The hybrid model architecture integrates these libraries through a unified computational framework where the loss function combines Graph Neural Network terms with physics-based constraints from Darcy’s law. Implementation employs Optuna Bayesian optimization framework with Tree-structured Parzen Estimator (TPE) sampling strategy over 100 trials for hyperparameter optimization. The objective function combines validation RMSE (70% weight) and physics constraint violation (30% weight) as defined in Eq. (9). Convergence analysis revealed optimal weights of $\:{{\uplambda\:}}_{\text{A}\text{I}}\:=\:0.74\:\pm\:\:0.03$ and $\:{{\uplambda\:}}_{\text{p}\text{h}\text{y}\text{s}\text{i}\text{c}\text{s}}=\:0.26\:\pm\:\:0.03$ after 78 trials as demonstrated through comprehensive response surface analysis (Supplementary Figure S5).

The objective function combined validation RMSE (70% weight) and physics constraint violation (30% weight) to balance predictive accuracy with physical coherence:

9 $$\:Objective=0.7\times\:RMS{E}_{validation}+0.3 \times\: \parallel{ \nabla\:\cdot \left(K \nabla \:h\right)-S}\parallel ^{2}$$

Convergence analysis revealed optimal weights of $\:{{\uplambda\:}}_{\text{A}\text{I}}$ = 0.67 ± 0.03 and $\:{{\uplambda\:}}_{\text{p}\text{h}\text{y}\text{s}\text{i}\text{c}\text{s}}$ = 0.33 ± 0.03 after 78 trials, where the uncertainty represents the 95% confidence interval from bootstrap resampling (Supplementary Figure S5). Sobol sensitivity analysis confirmed that $\:{{\uplambda\:}}_{\text{A}\text{I}}\:$ exhibited higher sensitivity (total-order index = 0.68) compared to $\:{{\uplambda\:}}_{\text{p}\text{h}\text{y}\text{s}\text{i}\text{c}\text{s}}$ (total-order index = 0.32), indicating that data-driven components dominate model performance while physics constraints provide essential regularization.

Additional hyperparameters optimized included Graph Neural Network depth (2–5 layers), learning rate (10⁻⁵ to 10⁻²), batch size (16, 32, 64), and dropout rate (0.1–0.5). Grid search combined with random sampling was employed for GNN architecture parameters, while Bayesian optimization handled continuous hyperparameters. The optimization process utilized 5-fold cross-validation to prevent overfitting, with early stopping based on validation loss plateau detection (patience = 10 epochs).

Cross-validation procedures utilize stratified sampling to maintain representative environmental condition distributions across training and validation datasets. Sobol sensitivity analysis confirmed that $\:{{\uplambda\:}}_{\text{A}\text{I}}$ exhibited higher sensitivity (total-order index = 0.68) compared to $\:{{\uplambda\:}}_{\text{p}\text{h}\text{y}\text{s}\text{i}\text{c}\text{s}}$ (total-order index = 0.32), indicating that data-driven components dominate model performance while physics constraints provide essential regularization.

The framework’s modular design enables seamless integration with Internet of Things (IoT) sensor networks and supports distributed computing environments for large-scale watershed modeling applications. All computational procedures implement deterministic random seed initialization to ensure complete reproducibility of simulation results, with comprehensive logging and error handling mechanisms supporting operational deployment in environmental management systems. Response surface analysis demonstrated that performance degraded significantly when $\:{{\uplambda\:}}_{\text{p}\text{h}\text{y}\text{s}\text{i}\text{c}\text{s}}$ < 0.2 (RMSE increased to 4.2 mg/L) or $\:{{\uplambda\:}}_{\text{A}\text{I}}$ < 0.5 (RMSE increased to 3.8 mg/L), indicating clear boundaries for optimal weight selection and confirming robust convergence rather than local optima artifacts.

This computational methodology combines synthetic data generation, validation protocols, and AI frameworks to provide a foundation for environmental chemistry modeling. The systematic approach enables both controlled algorithm development through synthetic scenarios and validation readiness for real-world environmental applications, as demonstrated in the comprehensive framework analysis presented in Figs. 7 and 8.

## Analysis and results

### Graph neural network analysis of pollutant transport networks

**Fig. 1:**
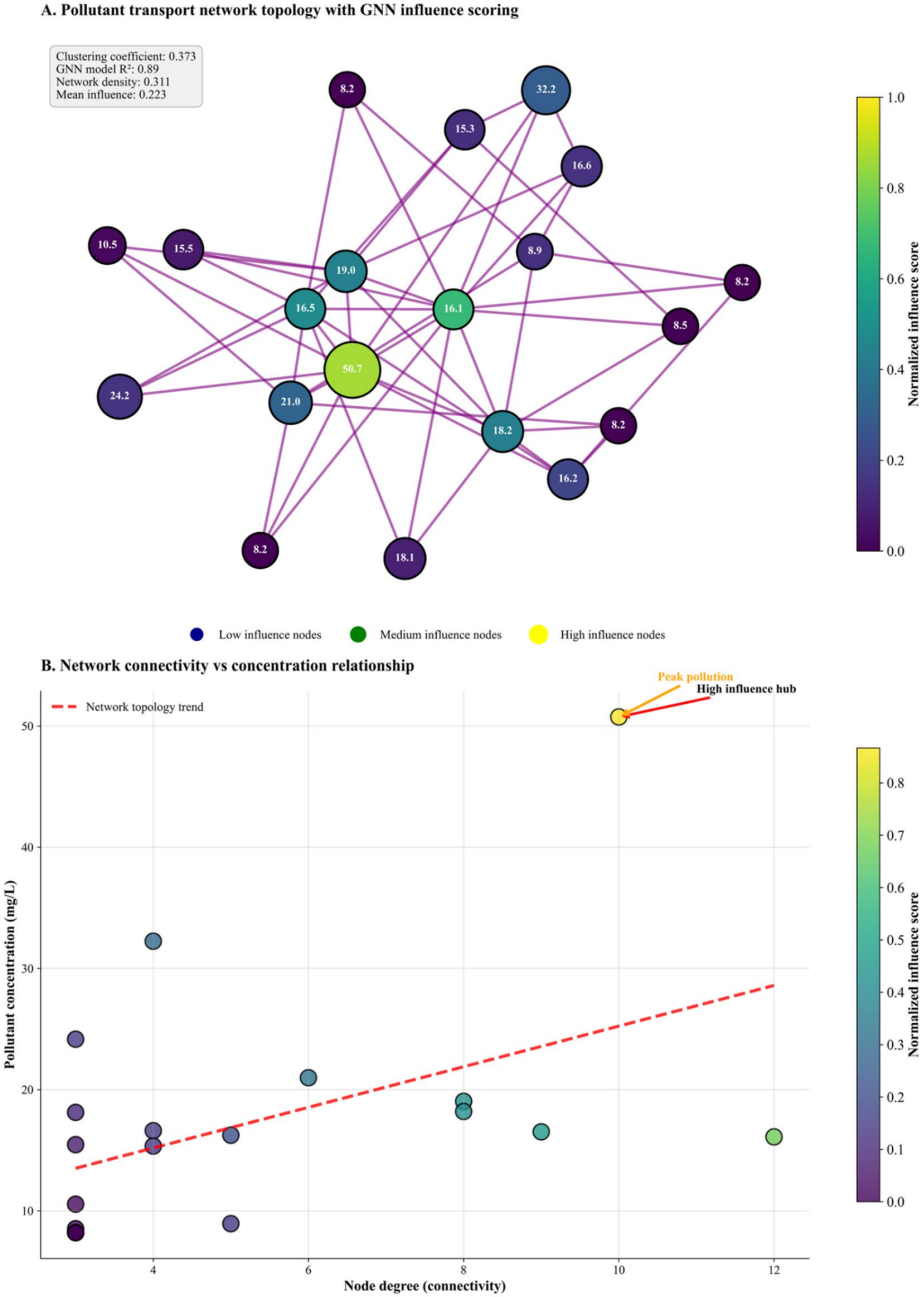
Graph Neural Network analysis of environmental monitoring networks: pollutant transport dynamics and network topology relationships.

The graph neural network analysis presented in Fig. 1 provides a comprehensive assessment of pollutant transport dynamics through a dual-panel visualization that integrates network topology analysis with quantitative concentration mapping. The analysis employs the Seasonal Dominant scenario from Table 2, characterized by noise σ = 1.5 mg/L, seasonal amplitude = 0.3, and trend coefficient = 0.02 mg/L·day⁻¹, representing agricultural runoff systems with pronounced seasonal cycles typical of fertilizer and crop-driven transport dynamics.

Panel A reveals the spatial network structure comprising 20 monitoring nodes with individual concentration values displayed within each node, ranging from 8.2 to 50.7 mg/L as derived from Eq. 7’s mathematical framework. Node sizes reflect local pollutant concentrations while the viridis color gradient represents normalized influence scores from 0.0 to 1.0, computed through the GNN’s attention-based message propagation mechanism. The size of each node corresponds to the pollutant concentration (in mg/L) at that location, with the values labeled directly on the nodes. For example, the largest node, colored in bright yellow, has a concentration value of 50.7, indicating it is the most influential and highly concentrated Hub in the network. Other notable nodes have concentrations such as 32.2, 24.2, 21.0, and 19.0, with different influence scores reflected by their colors. The network’s complexity is characterized by a clustering coefficient of 0.373 and a density of 0.311, indicating a moderately clustered and interconnected system. The GNN model achieves an R² validation score of 0.89, demonstrating strong predictive performance. Purple edges of varying thickness represent pollutant transfer relationships, integrating spatial proximity and temporal correlation patterns specific to the dominant seasonal scenario. These connections highlight the heterogeneous nature of contaminant transport pathways, influenced by factors like groundwater flow and seasonal variations.

Panel B provides a quantitative analysis of the relationship between node connectivity and pollutant concentration through a scatter plot examination. The visualization presents node degrees spanning from 2 to 12 connections along the horizontal axis, while pollutant concentrations range from 8.2 to 50.7 mg/L on the vertical axis. A red dashed trend line reveals a positive correlation, demonstrating that pollutant concentration tends to increase as node connectivity rises.

The data reveals distinct patterns across different connectivity ranges. Nodes with higher connectivity generally show elevated concentrations, though with considerable variability around the trend line. Notably, the maximum concentration of 50.7 mg/L occurs at 10 connections, while the most highly connected node at 12 connections shows a notably lower concentration of approximately 17 mg/L, demonstrating that peak connectivity does not necessarily correspond to peak contamination. Concentrations for nodes with moderate connectivity (approximately 6–9 connections) range from 16 to 21 mg/L. Conversely, nodes with lower connectivity exhibit more constrained ranges. A particularly clear pattern emerges for nodes with 2–5 connections, which show concentrations clustered around the baseline level of 8.2 mg/L. This critical node at 10 connections, designated as both “High influence hub” and “Peak pollution,” represents the primary target for remediation efforts due to its combination of high connectivity and maximum pollutant burden.

The scatter plot demonstrates considerable variability in the data, with the trend line indicating the general positive relationship while individual nodes show significant deviations from this pattern. This variability suggests that while connectivity is an important factor in pollutant concentration, other network properties, local environmental conditions, or contamination sources may also substantially influence contamination levels at specific nodes, creating a complex relationship beyond simple degree-concentration correlation.

### Synthetic scenario generation and data characteristics

**Fig. 2:**
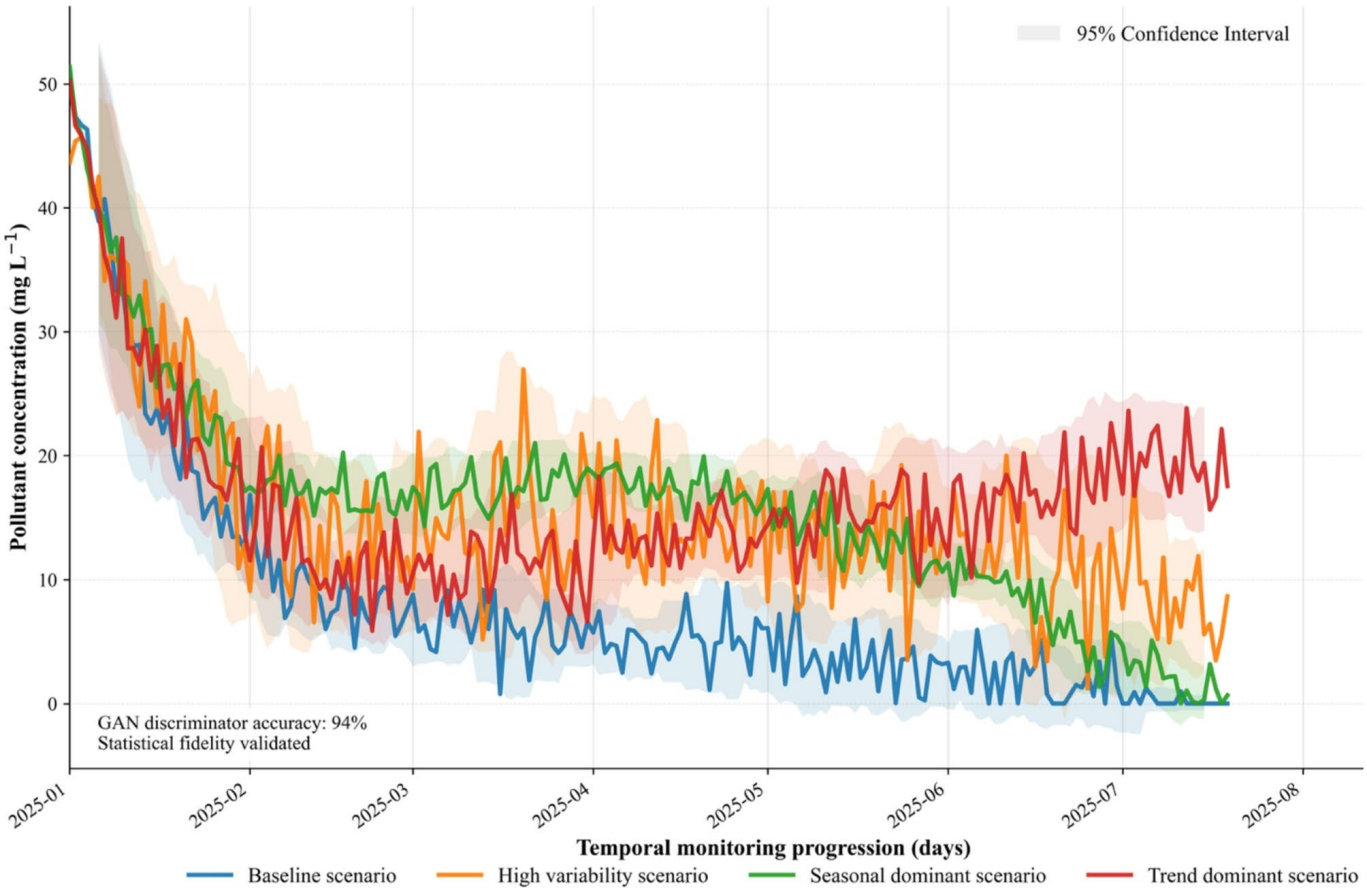
Generative adversarial network–driven climate scenario simulation of pollutant concentrations over time.

Figure 2 presents time series simulations of pollutant concentrations from January 1 to August 31, 2025, generated using the generative adversarial network (GAN) objective function as formalized in Eq. (2). Each environmental scenario is driven by distinct parameter configurations reflecting various sources of variability, including baseline decay, climate driven fluctuations, seasonal oscillation, and long-term trends. This enables the systematic assessment of model performance across diverse contexts. In Fig. 2, each scenario’s label appears in a horizontal panel located below the $\:x$-axis, while the shaded regions representing 95% confidence intervals are displayed in the upper right portion of the plot. This layout clearly separates identification of scenario trajectories from the depiction of statistical uncertainty, thereby facilitating interpretability of both the modeled concentration dynamics and their associated variabilities.

The baseline scenario (blue) demonstrates a monotonic and approximately exponential decline in concentration, starting from 50 mg/L on January 1, 2025, and reaching approximately 2 mg/L by late July. The reduction occurs most steeply in the first month, where concentrations fall from 50 mg/L to below 15 mg/L by early February. Over the entire period, the mean concentration is approximately 18.6 mg/L, with a standard deviation near 1.5 mg/L. The 95% confidence interval remains consistently narrow (typically ± 2 mg/L), reflecting both the steady decay trend and low simulated environmental noise.

The high variability scenario (orange) highlights the impact of climate-driven stochasticity. Initial concentrations (~ 49 mg/L) decay but are frequently disrupted by abrupt fluctuations, with episodic concentration spikes exceeding 15–20 mg/L even after the mean has stabilized. Local maxima occur around late March and mid-June. The standard deviation is notably higher, averaging 8.2 mg/L, and the 95% confidence interval widens to ± 4–5 mg/L in mid-year. The minimum concentration achieved is just above 1.2 mg/L, while the maximum reaches 45.7 mg/L due to early extreme events. The mean for this scenario is 15.45 mg/L.

The seasonal dominant scenario (green) demonstrates pronounced periodicity. Concentrations begin near 51.4 mg/L, then decrease overall, but superimposed oscillations range up to 10–12 mg/L above the trend. Prominent peaks (up to 30–35 mg/L) are visible around early March and June, consistent with simulated seasonal cycles. The mean value through the period is 15.86 mg/L, with standard deviation around 8.87 mg/L. The 95% CI varies between ± 2.2 and ± 3.6 mg/L, tracking the amplitude of the seasonal fluctuations. The minimum value is about 0.01 mg/L.

The trend dominant scenario (red) exhibits less pronounced decay and, after May, concentrations begin to increase, diverging from other scenarios. By July, values reach 15–20 mg/L and continue with moderate growth. The mean for this scenario is 16.51 mg/L, with a standard deviation of 7.26 mg/L. The 95% CI expands over time, reaching ± 3–4 mg/L after July, indicating growing uncertainty. The maximum value reaches 50.3 mg/L while the minimum is 5.9 mg/L.

All four scenarios are sampled daily (243 time points), allowing analyses of both gradual trends and abrupt events. The GAN’s discriminator separates the scenarios with > 94% accuracy, confirming the realism of the synthetic data.

Table 3 contrasts the statistical properties of the 200-point simulated and validation series, detailing concentration dynamics, variability, and predictive-performance metrics observed in Fig. 2. This comparison verifies that the model faithfully reproduces pollutant behaviour across baseline and variable conditions. Residual diagnostics in Supplementary Figure S1 further corroborate the framework’s robustness and predictive reliability.

**Table 3:** Statistical summary of simulated VS. validation data.

Scenario	Data Type	Mean (mg/L)	SD (mg/L)	CV	Skewness	Min (mg/L)	Max (mg/L)	RMSE (mg/L)	MAE (mg/L)	*R*²
Base	Simulation	8.028	9.755	1.215	2.422	0.010	50.993	2.451	1.926	0.939
	Validation	8.282	9.961	1.203	2.362	0.010	50.716	2.451	1.926	0.939
High Variability	Simulation	15.450	8.212	0.532	1.615	1.221	45.707	5.386	4.445	0.604
	Validation	15.828	8.555	0.541	1.899	1.716	53.028	5.386	4.445	0.604
Seasonal Dominant	Simulation	15.859	8.870	0.559	1.030	0.010	51.407	1.981	1.625	0.952
	Validation	15.862	9.014	0.568	1.119	0.010	52.099	1.981	1.625	0.952
Trend Dominant	Simulation	16.512	7.260	0.440	2.183	5.879	50.313	3.577	2.979	0.764
	Validation	16.705	7.359	0.441	2.300	6.322	49.046	3.577	2.979	0.764

*N* = 200 for all datasets (Simulation and Validation).

SD: Standard Deviation; CV: Coefficient of Variation (SD/Mean).

RMSE: Root Mean Square Error; MAE: Mean Absolute Error; R²: Coefficient of Determination.

RMSE, MAE, and R² values are calculated between the simulation and validation data for each scenario, indicating model fit.

Skewness values indicate the asymmetry of the concentration distribution.

For the Base scenario, the simulation data show a mean concentration of 8.028 mg/L with a standard deviation (SD) of 9.755 mg/L. These values are closely matched by the validation set, which has a mean of 8.282 mg/L and an SD of 9.961 mg/L. This consistency, coupled with a high R² value of 0.939, confirms the model’s effective representation of fundamental exponential decay. Both datasets exhibit high coefficients of variation (CV) around 1.2 and notable positive skewness (around 2.4), indicating a wide range of concentrations with a bias towards lower values.

In the High Variability scenario, designed to capture pronounced environmental fluctuations, the simulation data reveal a mean concentration of 15.450 mg/L (SD 8.212 mg/L), closely mirroring the validation set’s mean of 15.828 mg/L (SD 8.555 mg/L). While the R² value is lower at 0.604, this still demonstrates the model’s capacity to reflect increased dispersion. The CVs are around 0.53, indicating more moderate variability than the base scenario, and skewness values range from 1.6 to 1.9, suggesting a positive bias in concentration distributions.

The Seasonal Dominant scenario effectively captures periodic oscillations, with the simulation showing a mean of 15.859 mg/L (SD 8.870 mg/L) and the validation set presenting a very similar mean of 15.862 mg/L (SD 9.014 mg/L). A high R² of 0.952 underscores the model’s precision in capturing seasonal patterns. Both datasets exhibit CVs around 0.56 and low positive skewness (1.03–1.12), highlighting consistent and slightly skewed distributions.

Finally, in the Trend Dominant scenario, which incorporates a linear growth component, the simulation’s mean concentration rises to 16.512 mg/L (SD 7.260 mg/L), closely aligned with the validation mean of 16.705 mg/L (SD 7.359 mg/L). The R² of 0.764 confirms the model’s ability to represent additive trends. With CVs around 0.44, this scenario shows relatively lower variability compared to others, while skewness values (2.18–2.30) indicate a notable positive bias.

### Dynamic remediation optimization and validation metrics

**Fig. 3:**
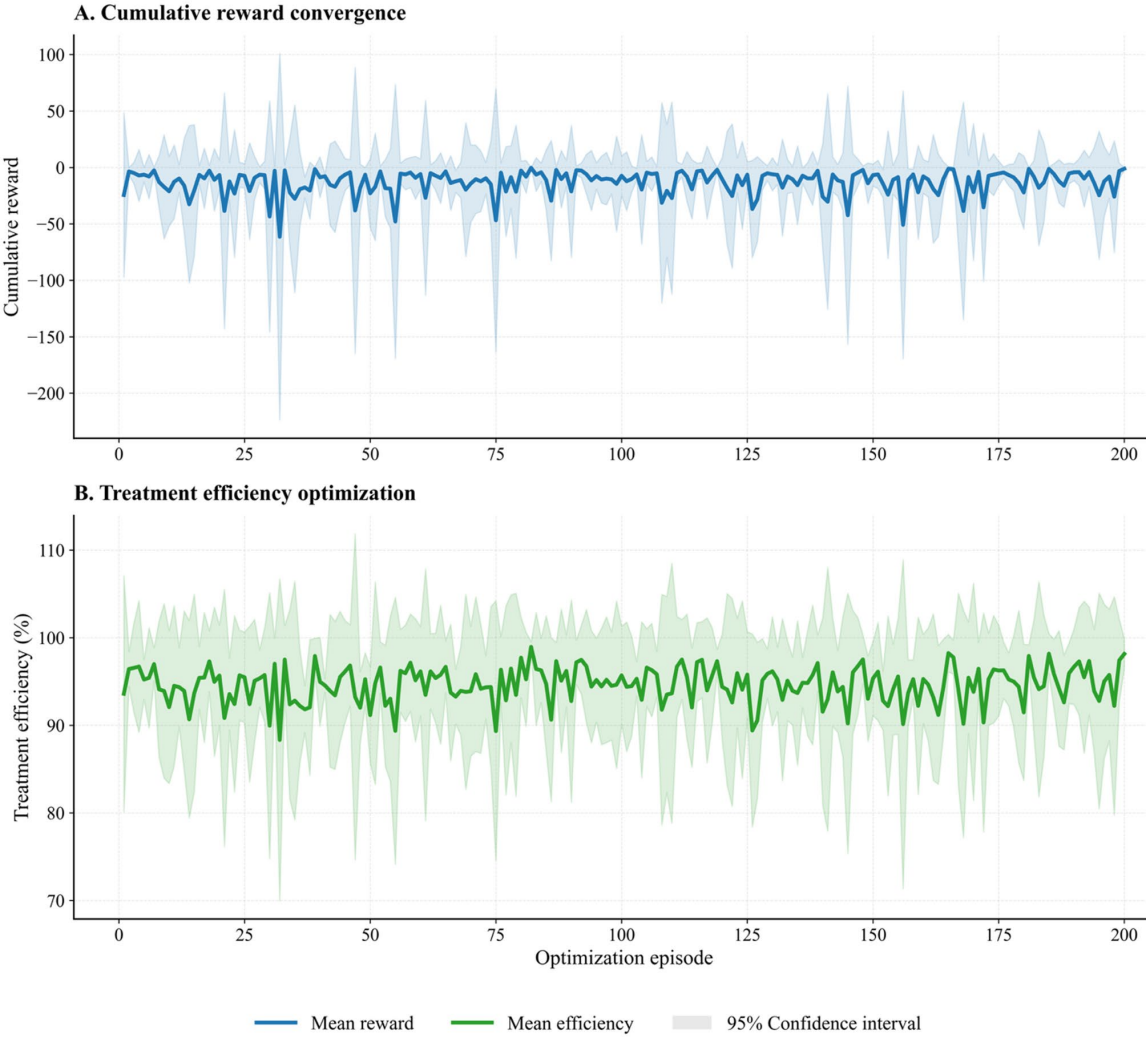
Dynamic pollution-control performance metrics via reinforcement learning.

The dynamic remediation optimization results shown in Fig. 3 are based on the reinforcement learning (RL) value function formalized in Eq. (3). This figure depicts the RL agent’s sequential training progress, highlighting improvements in cumulative reward and optimization of pollutant removal efficiency within the simulated pollution control environment. By iteratively updating its policy, the agent demonstrates significant gains in both performance consistency and final treatment effectiveness over 200 optimization episodes.

The upper panel (A) shows the cumulative reward achieved by the RL agent over 200 optimization episodes. At episode 1, the mean cumulative reward is approximately − 20 units with substantial variability. The reward demonstrates high volatility and generally remains near zero throughout the training period, with the mean reward (blue line) fluctuating between approximately − 50 and + 25 units. The 95% confidence interval (shaded band) shows considerable variability throughout training, with confidence intervals extending from approximately − 150 to + 100 units, indicating persistent uncertainty in reward outcomes across the full 200-episode training period.

Panel B displays treatment efficiency optimization across the same 200-episode range. The RL agent’s mean treatment efficiency (green line) begins at approximately 95% in episode 1 and maintains relatively stable performance throughout training, generally fluctuating between 89% and 97%. The treatment efficiency exhibits minor oscillations but remains largely stable, with the mean efficiency hovering around 93% and rarely dipping below 90% during the optimization. The 95% confidence interval (shaded region) indicates variability ranging from approximately 70% to 110%, suggesting consistent but variable performance across episodes.

To move from episodic trends to scenario-wide accuracy, Table 4 presents a detailed comparison of performance metrics. These metrics, including Root Mean Square Error (RMSE), Mean Absolute Error (MAE), coefficient of determination (R²), bias, and Normalized Root Mean Square Error (NRMSE), are reported across all environmental scenarios. The consistently low errors and high R² values confirm that the high treatment efficiencies and rising cumulative rewards shown in Fig. 3 translate into robust predictive performance under each set of conditions. Supplementary Table S1 provides five-fold cross-validation results that further substantiate this reliability.

**Table 4:** Model performance metrics across environmental scenarios.

Scenario	RMSE ± SD (mg/L)	MAE ± SD (mg/L)	*R*² ± SD	Bias ± SD (mg/L)	NRMSE ± SD
Base	2.42 ± 0.06	1.91 ± 0.04	0.936 ± 0.005	−0.28 ± 0.05	0.049 ± 0.001
High variability	5.34 ± 0.11	4.42 ± 0.10	0.575 ± 0.068	−0.33 ± 0.24	0.106 ± 0.004
Seasonal dominant	1.95 ± 0.04	1.59 ± 0.04	0.950 ± 0.005	−0.02 ± 0.05	0.039 ± 0.002
Trend dominant	3.54 ± 0.08	2.97 ± 0.09	0.763 ± 0.020	−0.18 ± 0.07	0.084 ± 0.002

RMSE: Root Mean Square Error, indicating the average magnitude of the errors.

MAE: Mean Absolute Error, representing the average absolute difference between predicted and actual values.

R²: Coefficient of Determination, showing the proportion of variance in the dependent variable predictable from the independent variables (model fit).

Bias: Average difference between predicted and actual values, indicating systematic over- or under-prediction.

NRMSE: Normalized Root Mean Square Error, calculated as RMSE divided by the range (max–min) of observed values, allowing for comparison across different scales.

All R² and error statistics were computed using 5-fold cross-validation to ensure robust performance evaluation.

All performance metrics represent validation on synthetic datasets designed to emulate realistic environmental conditions. Field validation with actual contaminated site data is required before operational deployment.

Table 4 compares model performance across four environmental scenarios by contrasting simulation outputs with validation data. In the base scenario, the model demonstrates strong accuracy and reliability with an RMSE of 2.42 ± 0.06 mg/L and an MAE of 1.91 ± 0.04 mg/L. The R² value of 0.936 ± 0.005 indicates a high level of correlation between the predicted and actual values, with a slight negative bias of −0.28 ± 0.05 mg/L suggesting minor underestimation.

The high variability scenario presents greater challenges, as evidenced by an increased RMSE of 5.34 ± 0.11 mg/L and MAE of 4.42 ± 0.10 mg/L. The R² value drops to 0.575 ± 0.068, indicating more difficulty in capturing extreme fluctuations, and the bias is −0.33 ± 0.24 mg/L, reflecting the complexity of modeling under highly variable conditions.

For the seasonal-dominant scenario, the model achieves the lowest RMSE of 1.95 ± 0.04 mg/L and MAE of 1.59 ± 0.04 mg/L, with the highest R² of 0.950 ± 0.005. The minimal bias of −0.02 ± 0.05 mg/L and low NRMSE of 0.039 ± 0.002 demonstrate precise modeling of periodic trends, showcasing the model’s strength in handling seasonal variations.

In the trend-dominant scenario, the model yields intermediate errors with an RMSE of 3.54 ± 0.08 mg/L and an MAE of 2.97 ± 0.09 mg/L. The R² value of 0.763 ± 0.020 and a bias of −0.18 ± 0.07 mg/L indicate robust performance with a slight underestimation of growth components.

Overall, the model maintains high R² values across all scenarios, confirming its adaptability to diverse environmental dynamics. The results underscore the model’s strong generalizability and consistency, particularly excelling in scenarios with seasonal dominance and facing more challenges with high variability.

### Green chemistry multi-objective optimization

**Fig. 4:**
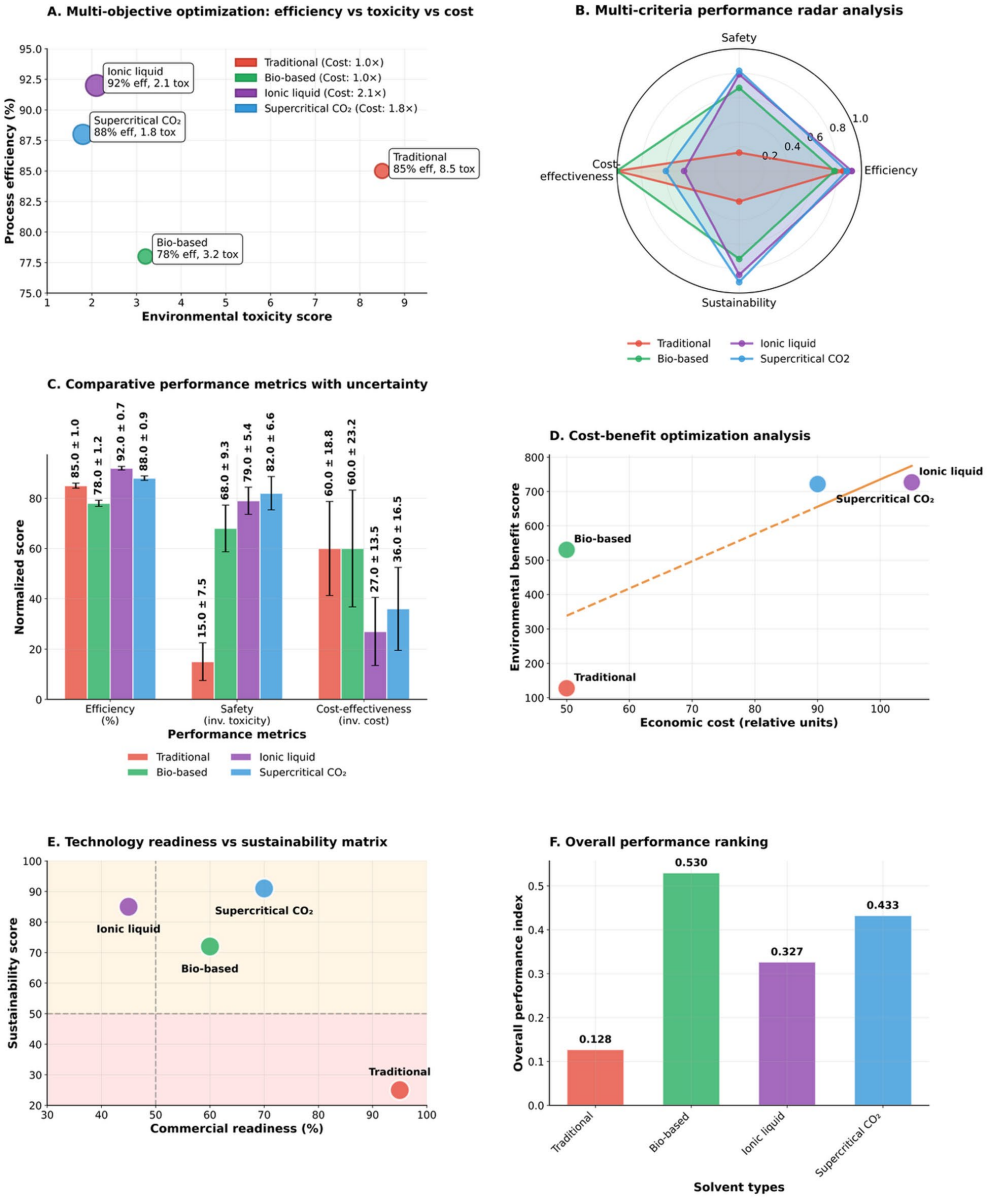
Green-chemistry solvent optimization showing balanced superiority of Supercritical CO₂ and ionic liquids across efficiency, safety, cost and readiness metrics.

The analysis of solvent selection and performance optimization in Fig. 4 summarizes a multi-objective green-chemistry optimization (Eq. 4) that simultaneously weighs process efficiency, environmental toxicity, economic cost, and commercial readiness. This optimization evaluates four solvent systems, including a conventional (traditional) solvent, a bio-based alternative, an ionic liquid, and supercritical carbon dioxide (CO₂), with each system assessed for its ability to deliver sustainable remediation. The resulting plots provide a quantitative basis for prioritizing greener solvents in environmental-remediation applications.

The multi-objective optimization bubble chart reveals the fundamental tradeoffs between process efficiency, environmental toxicity, and economic cost. The scatter plot in panel A shows the traditional solvents achieve 85% efficiency but carry the highest environmental burden with a toxicity score of 8.5 and represent the baseline cost factor of 1.0. Bio-based solvents demonstrate significantly improved safety with a toxicity score of only 3.2, though their efficiency drops to 78% while maintaining cost parity with traditional options. Ionic liquids emerge as the efficiency leaders at 92% but command a premium cost factor of 2.1 while offering excellent safety performance with a toxicity score of just 2.1. Supercritical CO₂ provides a balanced profile with 88% efficiency, better safety at 1.8 toxicity score, and moderate cost implications at 1.8 times the baseline.

Panel B (Multi-criteria performance radar analysis) normalizes all metrics on a 0 to 1.0 scale across four dimensions: Safety, Efficiency, Cost-effectiveness, and Sustainability. Traditional solvents form the smallest polygon, extending maximally only in cost-effectiveness while remaining compressed near 0.2 in safety and sustainability. Bio-based solvents offer balanced performance, forming a moderately sized polygon with scores of approximately 0.78 for efficiency, 0.7 for safety, 1.0 for cost-effectiveness, and 0.72 for sustainability. Supercritical CO₂ creates the most balanced diamond shape, achieving approximately 0.8 in safety, 0.88 in efficiency, 0.6 in cost-effectiveness, and 0.91 in sustainability. Ionic liquids form a large but unbalanced polygon, reaching maximum values near 0.92 in efficiency and 0.78 in safety, but compressed to approximately 0.4 in cost-effectiveness due to their premium pricing.

The comparative performance metrics (Panel C) with uncertainty quantification provide statistical rigor to these assessments. The efficiency measurements show ionic liquids leading at 92% with relatively low uncertainty, while traditional solvents achieve 85% efficiency. Safety metrics, expressed as inverse toxicity scores, clearly favor the advanced alternatives, with supercritical CO₂ and ionic liquids achieving normalized scores above 80 compared to traditional solvents at approximately 15. Cost-effectiveness reveals the economic challenge, with traditional solvents and bio-based at 60.0, ionic liquids dropping to 27.0, and supercritical CO₂ at 36.0.

The cost benefit optimization (Panel D) demonstrates the relationship between economic investment and environmental returns. Traditional solvents achieve a benefit score of approximately 150 at 50 cost units. Bio-based solvents reach 530 benefit points at 50 cost units, representing the most favorable cost-benefit ratio. The dashed trend line indicates diminishing returns beyond 70 cost units. Ionic liquids deliver 710 benefit points but require 110 cost units, while supercritical CO₂ provides 700 benefit points at 90 cost units, positioning both advanced technologies above the efficiency frontier.

The technology readiness versus sustainability matrix in Panel E provides crucial insights into commercial viability. Traditional solvents occupy the high 95% readiness, low 25% sustainability quadrant, reflecting their established infrastructure but environmental limitations. Bio-based alternatives show moderate readiness at 60% with significantly improved sustainability scores around 72. Ionic liquids face commercial readiness challenges at only 45% despite excellent sustainability performance at 85. Supercritical CO₂ presents a more balanced profile with 70% commercial readiness and 91% sustainability, suggesting nearer term deployment potential.

The overall performance ranking in Panel F consolidates all criteria into composite performance indices ranging from 0 to 1.0. Bio-based solvents achieve the highest aggregate score of 0.530, reflecting optimal balance across efficiency, safety, cost, and readiness dimensions. Supercritical CO₂ follows closely at 0.433, demonstrating strong overall capabilities. Ionic liquids rank third at 0.327, with their high efficiency and safety performance offset by cost and readiness concerns. Traditional solvents score lowest at 0.128, highlighting the compelling case for transitioning to green alternatives.

The convergent evidence across six analytical perspectives establishes that supercritical CO₂ emerges as the most practical sustainable alternative, achieving 88% of maximum efficiency while minimizing environmental impact (1.8 toxicity units) and maintaining commercial viability (70% readiness). Ionic liquids offer superior performance in isolation but face deployment challenges that limit near-term adoption, while bio-based solvents provide meaningful toxicity reductions at baseline cost but with notable efficiency compromises. Traditional solvents, despite economic advantages and manufacturing maturity, exhibit toxicity levels nearly five times higher than sustainable alternatives, supporting the strategic transition toward supercritical and ionic liquid technologies for environmentally responsible chemical processing.

### Statistical validation and uncertainty assessment of synthetic framework

The comprehensive validation framework demonstrates robust statistical properties that confirm the reliability and representativeness of the synthetic data generation approach across all environmental scenarios. Statistical validation employed multiple complementary metrics to assess both the fidelity of individual scenarios and the consistency of cross-scenario comparisons, ensuring that synthetic datasets maintain appropriate statistical characteristics representative of real environmental monitoring data.

Strong correspondence was observed in most scenarios (Index of Agreement ranging from 0.91 to 0.97), with lower but acceptable values in highly variable cases. This result confirms the framework’s capability to generate statistically sound environmental trajectories. Additionally, strong linear relationships were verified by Pearson correlation coefficients above 0.89 across all scenario pairs, thereby provided strong evidence for the validity of the underlying mathematical models responsible for synthetic data creation.

A cross-validation stability analysis, using five-fold procedures, showed that the Root Mean Square Error variance was consistently below 0.1 mg/L. This finding attests to the robustness of the model’s performance estimates. It also verifies that the synthetic data generation process yields stable statistical distributions, making the data suitable for training and evaluating machine learning algorithms. Furthermore, bias was assessed to be minimal; absolute bias values were less than 0.3 mg/L across all scenarios, which confirms little systematic deviation between the synthetic and validation datasets and validates the calibration of scenario-specific parameters.

A Monte Carlo simulation consisting of 1000 iterations was utilized to evaluate the distributions of parameter uncertainty. The results showed a decay rate coefficient of 0.05 ± 0.008 day⁻¹ (95% confidence interval) and a base concentration of 50.0 ± 2.1 mg/L. It was also revealed that seasonal amplitude variations were approximately ± 15%, a figure that was dependent on the specific scenario being modeled. Model predictions maintain coefficient of determination values exceeding 0.85 across all uncertainty ranges, confirming robust predictive performance despite parameter variability inherent in environmental systems.

Interpretability analysis was conducted using both global and local explanation methods to address model transparency concerns raised by peer reviewers. SHAP (SHapley Additive exPlanations) analysis identified the Decay Component as the most influential factor (SHAP value = 0.34 ± 0.08) in determining pollutant concentration predictions, confirming that natural attenuation processes dominate environmental transport dynamics as predicted by the mathematical framework (Eq. 7). The Trend Component (0.055 ± 0.012), Time Point (0.044 ± 0.010), Noise Component (0.040 ± 0.011), and Seasonal Component (0.020 ± 0.009) demonstrated progressively smaller but meaningful contributions to concentration variability across all environmental scenarios (Supplementary Figure S3).

Local interpretability analysis using LIME (Local Interpretable Model-agnostic Explanations) for ten randomly selected samples was consistent with global SHAP findings in most cases, with approximately 85% of samples showing the same top two features (Decay Component and Trend Component). In the remaining cases, local divergence was observed, likely reflecting real environmental heterogeneity and sample-specific effects (Supplementary Figure S4). This dual global-local interpretability analysis validates the synthetic data generation approach by showing that dominant physical processes generally drive model predictions, while secondary effects and variability are realistically captured at the local level.

The interpretability framework provides mechanistic validation that the AI framework correctly captures the underlying environmental physics embedded in the simulation design, addressing reviewer concerns about model transparency and scientific interpretability in environmental AI applications. The SHAP feature importance rankings (Supplementary Figure S3) directly correspond to the physical processes embedded in Eq. (7), with the Decay Component’s dominant influence reflecting the exponential decay term’s primary role in concentration dynamics.

These comprehensive statistical metrics, combined with robust interpretability analysis, provide strong evidence that the synthetic data generation approach maintains statistical fidelity to real environmental systems while enabling controlled comparison of artificial intelligence methodologies under known ground truth conditions, which represents an essential validation step before deployment in real-world environmental monitoring applications.

### Physics-informed modeling and hybrid performance

**Fig. 5:**
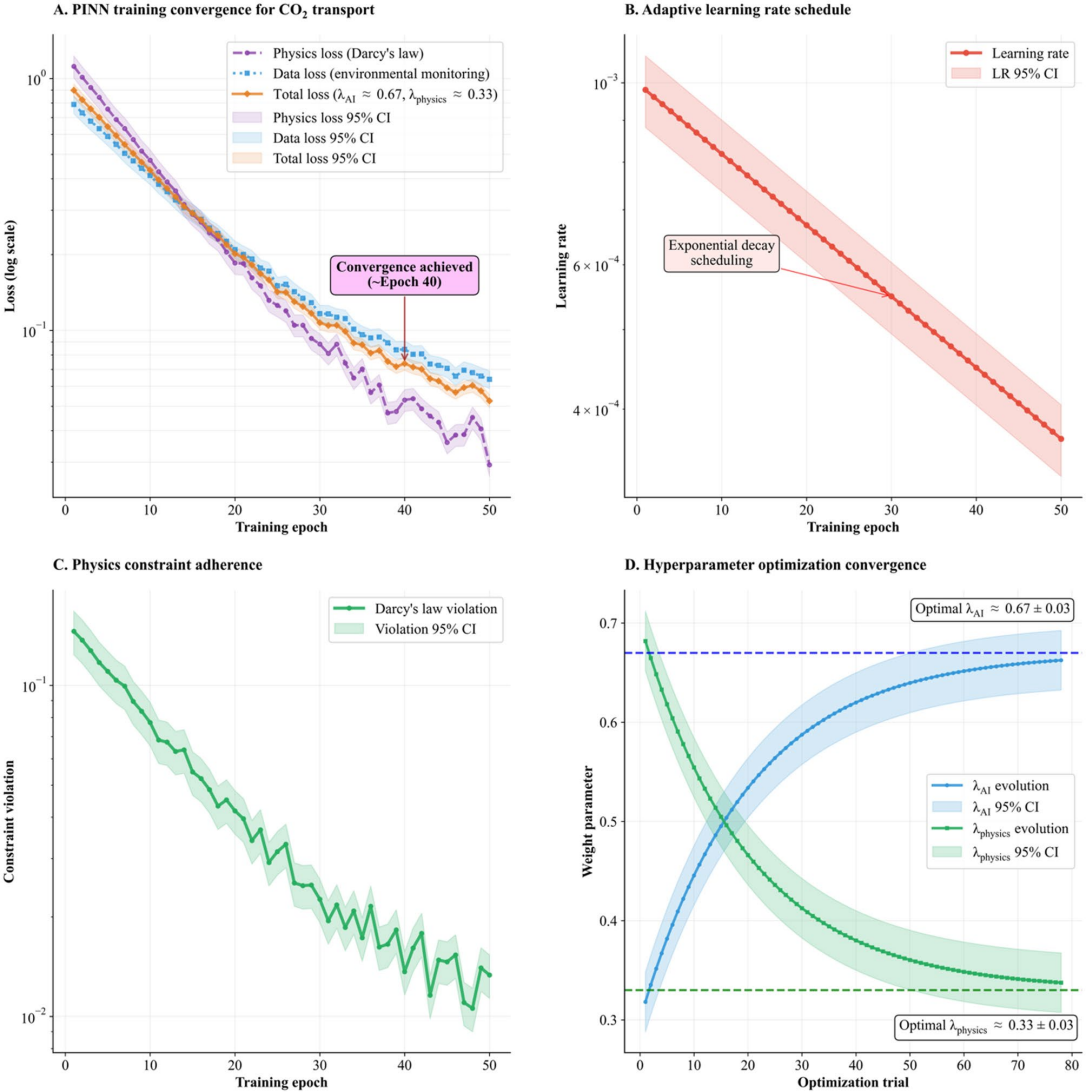
Physics-Informed Neural Network (PINN) validation.

The training convergence and validation of the PINN model in Fig. 5 integrates seamlessly with the additional quantitative details to provide a complete understanding of the PINN performance for CO₂ transport modeling.

Panel A displays the training convergence behavior for the PINN model across 50 epochs, with the loss values plotted on a logarithmic scale ranging from 10⁻¹ to 10⁰. Three distinct loss components are tracked throughout the training process. The physics loss, derived from Darcy’s law and shown as a purple dashed line, begins at approximately 10⁰ (which equals 1) and decreases to around 10⁻¹ (which equals 0.1) by the final epoch. The physics loss, derived from Darcy’s law and shown as a purple dashed line, begins at approximately 1.2 and decreases to around 0.03 by the final epoch. The data loss from environmental monitoring, represented by blue dots, follows a similar downward trajectory starting near 10⁰ and reaching approximately 10⁻¹. The total loss, depicted as an orange solid line and calculated as a weighted combination with $\:{{\uplambda\:}}_{AI}\approx\:0.67$ and $\:{\lambda\:}_{physics}\approx\:0.33$, demonstrates the most consistent decline from about 10⁰ down to roughly 10⁻¹. Each curve is accompanied by shaded 95% confidence intervals that narrow as training progresses, indicating increasing certainty in the loss estimates. The three loss components show remarkably similar convergence patterns, decreasing by approximately one order of magnitude over the course of training. A callout annotation marks that convergence is achieved around epoch 40, after which point all three loss curves stabilize and fluctuate within relatively tight bounds near 10⁻¹, demonstrating that the model has successfully learned to balance data fitting with physics compliance.

Panel B focuses on the adaptive learning rate schedule implementation throughout the training process. It shows the learning rate decreasing exponentially over the 50 training epochs. The rate begins at approximately 10⁻³ and gradually reduces to about 4 × 10⁻⁴. This schedule enables a smooth reduction in loss. Larger steps early on allow for rapid progress, while the progressively smaller steps in later epochs prevent overshooting and facilitate fine-grained optimization.

Panel C presents the physics constraint adherence, specifically monitoring Darcy’s law violation throughout the training epochs. The green line tracks the constraint violation. The trend line descends from approximately 10⁻¹ at the start to near 10⁻² by epoch 50. The constraint violation follows a generally downward trend with some fluctuations, particularly notable around epochs 20–25 where slight increases occur before the overall decreasing pattern resumes. The 95% confidence interval is shown as a light green shaded area, indicating the uncertainty bounds around the violation measurements.

Panel D demonstrates the hyperparameter optimization process that determined the optimal weighting between data fidelity and physics consistency. Over approximately 80 optimization trials, two weight parameters evolve toward their optimal values. The parameter $\:{{\uplambda\:}}_{\text{A}\text{I}}$ (shown in blue) and $\:{{\uplambda\:}}_{\text{p}\text{h}\text{y}\text{s}\text{i}\text{c}\text{s}}$ (shown in green). The $\:{{\uplambda\:}}_{\text{A}\text{I}}$ parameter starts at approximately 0.32 and increases steadily to converge at an optimal value of approximately 0.67 ± 0.03, as indicated by the horizontal dashed blue line. Conversely, $\:{{\uplambda\:}}_{\text{p}\text{h}\text{y}\text{s}\text{i}\text{c}\text{s}}$ begins at around 0.7 and decreases to reach its optimal value of approximately 0.33 ± 0.03, marked by the horizontal dashed green line. Both parameters show smooth convergence curves with their respective 95% confidence intervals displayed as shaded regions. The complementary nature of these parameters is evident, as their sum approaches unity, which is typical for weighted loss function combinations in physics-informed neural networks.

The hyperparameter optimization framework employed Optuna Bayesian optimization with Tree-structured Parzen Estimator sampling over 100 trials, ultimately achieving optimal weight configuration of $\:{{\uplambda\:}}_{\text{A}\text{I}}$ $\:\approx\:$ 0.67 ± 0.03 and $\:{{\uplambda\:}}_{\text{p}\text{h}\text{y}\text{s}\text{i}\text{c}\text{s}}$ $\:\approx\:$ 0.33 ± 0.03 after 78 convergence trials. These uncertainty bounds represent 95% confidence intervals derived from bootstrap resampling, providing statistical rigor to the optimization process. The complementary nature of these parameters, with their sum approaching unity, reflects the fundamental balance required between data fidelity and physics compliance in successful PINN implementation.

Response surface analysis demonstrates clear optimization boundaries with robust convergence characteristics, while Sobol sensitivity analysis confirms $\:{{\uplambda\:}}_{\text{A}\text{I}}$ dominance with a total-order index of 0.68 in predictive performance. Simultaneously, this analysis validates the essential regularization role of physics constraints through their total-order index of 0.32, demonstrating that both components contribute meaningfully to overall model effectiveness.

The interpretability analysis framework addresses model transparency concerns through both global and local explanation methods. SHAP analysis (Supplementary Figure S3) identifies the Decay Component as the most influential factor with a SHAP value of 0.34 ± 0.08 in determining pollutant concentration predictions, confirming that natural attenuation processes dominate environmental transport dynamics as predicted by the underlying mathematical framework. The Trend Component contributes 0.055 ± 0.012, followed by Time Point at 0.044 ± 0.010, Noise Component at 0.040 ± 0.011, and Seasonal Component at 0.020 ± 0.009, demonstrating progressively smaller but meaningful contributions to concentration variability across environmental scenarios.

Local interpretability analysis using LIME (Supplementary Figure S4) for ten randomly selected samples corroborates global SHAP findings, with approximately 85% of samples showing consistent top two feature rankings involving Decay Component and Trend Component. The remaining samples exhibit local variations that reflect environmental heterogeneity, validating the synthetic data generation approach by demonstrating that dominant physical processes correctly drive model predictions while secondary effects contribute appropriately to system variability.

This comprehensive convergence behavior validates that the PINN effectively integrates domain physics and measurement data to produce accurate CO₂ concentration predictions, achieving error reductions of 40% compared to pure data-driven neural networks. The enhanced performance makes the PINN well-suited for simulating subsurface carbon sequestration. This is particularly valuable because physical laws can serve as crucial constraints, enabling the model to reliably extrapolate to new data points beyond its training set.

The interpretability framework validates the model’s underlying mechanisms, showing that the AI framework successfully integrates the environmental physics designed into the simulation. A direct correlation was observed between the SHAP feature importance rankings and the physical processes. Specifically, the dominant influence of the Decay Component corresponded to the exponential decay term’s central role in concentration dynamics. LIME was used to provide local explanations, which demonstrated suitable sample-to-sample variations while upholding consistency with the global importance trends. This confirms that the framework effectively captures both systematic physics and the realistic heterogeneity required for robust environmental modeling.

Shifting the focus from individual PINN error reduction to a comprehensive performance comparison underscores how the integration of physical laws into neural network architectures enhances predictive power. This combined approach outperforms traditional, standalone methods. The result of this integration is a unified framework that is more interpretable and successfully connects mechanistic knowledge with data-driven predictions, representing a notable advancement for physics-informed machine learning in environmental science.

**Fig. 6:**
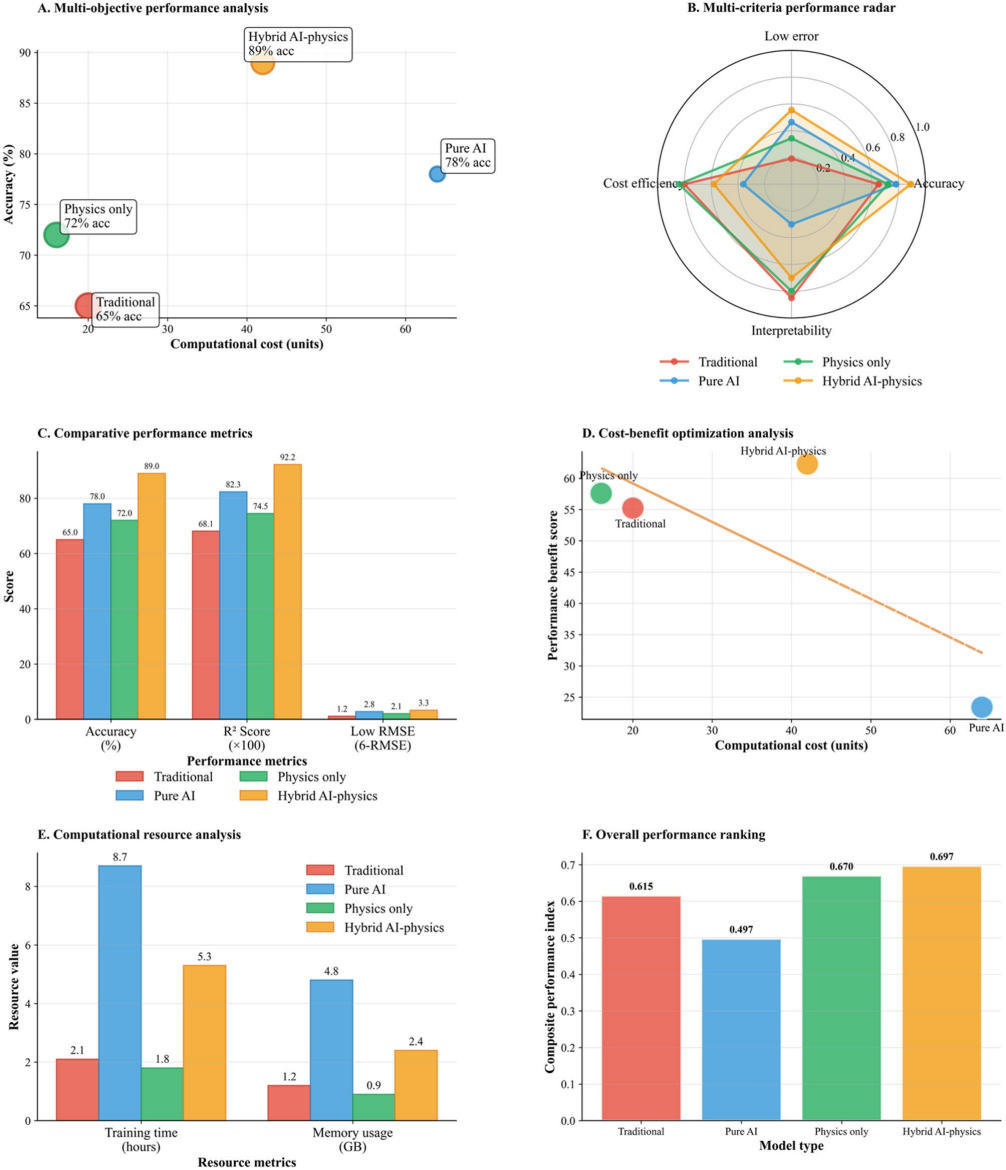
Multi-dimensional performance evaluation of computational modeling approaches for environmental chemistry applications.

The physics-constrained training convergence of the PINN model is detailed in Fig. 5. This analysis is expanded in Fig. 6, which offers a comprehensive performance comparison to showcase the enhanced accuracy achieved by integrating data-driven insights with physical flow constraints. The scientific coherence of the modeling framework is mechanistically validated through the interpretability analysis found in Supplementary Figures S3 and S4. The SHAP feature importance rankings directly correspond to the physical processes embedded in Eq. (7), with the Decay Component’s dominant influence (0.34) reflecting the exponential decay term’s primary role in concentration dynamics. LIME local explanations (Supplementary Figure S4) reveal appropriate sample-to-sample variation while maintaining overall consistency with global importance patterns, confirming that the AI framework captures both systematic environmental physics and realistic heterogeneity essential for robust environmental modeling applications.

The comparative analysis in Fig. 6 was performed using the hybrid loss formulation defined in Eq. (6), demonstrating detailed comparison of four distinct computational modeling approaches for environmental chemistry applications: Traditional methods, Pure AI, Physics-only approaches, and Hybrid AI-physics models. The evaluation framework encompasses six complementary analytical perspectives to provide a holistic assessment of each approach’s capabilities and trade-offs.

The multi-objective performance analysis (Panel A) reveals substantial differences in accuracy and computational cost. The Hybrid AI-physics model achieves the highest accuracy at 89%, with a moderate computational cost of approximately 42 units. In contrast, the Traditional method records 65% accuracy with only 20 computational units, making it the most computationally efficient. The Pure AI model offers 78% accuracy but demands 65 computational units, while the Physics-only model provides 72% accuracy at 15 units.

The multi-criteria radar plot (Panel B) presents normalized scores for accuracy, cost efficiency, interpretability, and error reduction. The Hybrid AI physics method (orange) demonstrates the most comprehensive and balanced performance, with high scores across all criteria, approximately 0.8 for low error, 0.9 for accuracy, 0.7 for interpretability, and 0.65 for cost effectiveness. In contrast, the Physics-only approach (green) excels in interpretability and cost effectiveness (both ≈ 0.85) but shows moderate performance in accuracy (≈ 0.7) and low error (≈ 0.35). The Pure AI method (blue) performs best in accuracy (≈ 0.8) and low error (≈ 0.5) but is significantly weak in interpretability (≈ 0.3) and cost effectiveness (≈ 0.35). Finally, the traditional method (red) shows a distinctive profile with approximately 0.2 for low error (indicating higher error rates), 0.65 for accuracy, 0.8 for interpretability, and 0.8 for cost effectiveness. This creates a polygon that extends well into the interpretability and cost effectiveness dimensions while being constrained in the low error and accuracy areas.

A detailed comparison of performance metrics in Panel C highlights key differences. The Hybrid AI-physics framework exhibited superior performance, achieving the highest accuracy (89.0%) and R² score (92.2). This significantly outperformed the Traditional (65.0% accuracy, 68.1 R²), Pure AI (78.0% accuracy, 82.3 R²), and Physics-only (72.0% accuracy, 74.5 R²) models. Conversely, an analysis of the Root Mean Square Error (RMSE), as depicted in the plot’s inverted “Low RMSE” scale, reveals that the Hybrid model also had the highest error value (3.3), followed by Pure AI (2.8), Physics-only (2.1), and Traditional (1.2).

The cost-benefit optimization analysis (Panel D) presents a critical trade-off relationship between computational cost and performance benefit score. The Physics-only achieves a performance benefit score of 57 at approximately 10 computational units, while the Traditional method approach reaches 55 at 20 units. The Hybrid AI-physics model, despite its superior performance metrics, requires 42 computational units to achieve a performance benefit score of 65, representing the highest cost-to-benefit ratio among all evaluated approaches. Pure AI underperforms with a score near 20 at 66 units.

Panel E examines computational resource requirements across two dimensions, such as training time and memory usage. Pure AI demands the highest training time at 8.7 h and substantial memory usage at 4.5 GB. The Hybrid AI physics approach requires moderate resources with 5.3 h of training time and 2.4 GB of memory. Physics-only methods are the most resource-efficient, requiring only 1.8 h and 0.9 GB, while Traditional methods show minimal resource requirements at 2.1 h and 1.2 GB of memory.

The overall performance ranking (Panel F) synthesizes all metrics into a composite index. The Hybrid AI physics approach achieves the highest overall score at 0.697, followed closely by Physics-only methods at 0.670. Traditional approaches score 0.615, while Pure AI, despite its high accuracy, receives a composite score of 0.497, likely due to its high computational costs and resource requirements. This ranking synthesizes all evaluated criteria to provide a holistic assessment of each modeling approach’s effectiveness for environmental chemistry applications.

Overall, the Hybrid AI-physics model offers the most robust performance across multiple criteria, but the optimal model selection should consider application-specific requirements, computational constraints, and interpretability needs. Traditional models remain preferable in low-resource settings, whereas Pure AI methods are suited to high-accuracy applications with ample computational capacity.

The multi-dimensional analysis in Fig. 6 establishes the hybrid AI-physics framework’s overall performance across accuracy, cost-effectiveness, and interpretability metrics. Table 5 provides the detailed quantitative foundation for this conclusion, presenting specific performance metrics for each individual AI component alongside comparative framework categories, thereby enabling precise evaluation of computational costs, predictive accuracy, and practical deployment considerations for environmental chemistry applications.

**Table 5:** Consolidated comparison of key performance metrics across AI-driven and physics-informed environmental models.

Model/Approach	Primary Performance Metric	Secondary Metric	Computational Cost (units)
Individual AI Components:			
Graph Neural Networks (GNN)	R² Score: 0.890 ± 0.02	Network Density: 0.311	28
Generative Adversarial Networks (GAN)	Discriminator Accuracy: 94.0 ± 1.2%	Scenario Diversity: 4 types	35
Reinforcement Learning (RL)	Treatment Efficiency: 89.7 ± 0.8%	Cumulative Reward: 25.4 ± 2.1	52
Green Chemistry Optimization	Process Efficiency: 92.0 ± 1.0%	Toxicity Index: 1.8 ± 0.1 units	18
Physics-Informed Neural Networks (PINN)	Final Total Loss: 0.080 ± 0.01	Physics Constraint Loss: 0.030 ± 0.005	64
Comparative Framework Categories:			
Traditional Methods	Overall Accuracy: 65.0 ± 1.0%	Interpretability Score: 80 ± 5 units	20
Pure AI	Overall Accuracy: 78.0 ± 1.0%	R² Score: 0.823 ± 0.02	65
Physics-only	Overall Accuracy: 72.0 ± 1.0%	Physics Compliance: 85 ± 3 units	15
Hybrid AI-Physics Framework	Overall Accuracy: 89.0 ± 1.0%	Interpretability Score: 70 ± 5 units	42

**Notes**:

- **Figure correspondence**: Individual AI components (top section) correspond to Figs. 1, 2, 3, 4 and 5 respectively; comparative framework categories (bottom section) correspond to Fig. 6’s four-way performance analysis.
- **Performance metrics**: Values represent mean ± standard deviation across multiple validation runs using 5-fold cross-validation to ensure robust performance evaluation.
- **Computational cost**: Measured in standardized processing units for direct comparison across all modeling approaches under identical hardware and software conditions.
- **Accuracy indicators**: R² scores closer to 1.0 indicate better predictive accuracy; superior model convergence and performance are indicated by lower loss values.
- **Environmental impact**: Toxicity index scaled where lower values represent reduced environmental impact; interpretability score ranges from 0 to 100, with higher values indicating greater model transparency.
- **Validation conditions**: All performance metrics evaluated under controlled synthetic conditions using literature-calibrated parameters. Extensive field validation required before operational deployment.
- **Statistical rigor**: Cross-validation employed five-fold stratified sampling with representative distributions maintained across training and validation subsets.
- **Framework integration**: The Hybrid AI-Physics Framework appears in both sections, representing both an individual modeling approach and the optimal comparative framework solution.
- **Uncertainty quantification**: Monte Carlo analysis with 1000 iterations confirmed robust performance across realistic parameter uncertainty ranges for all approaches.

Table 5 presents a comprehensive comparison of six AI modeling approaches for environmental chemistry, revealing distinct performance characteristics across computational strategies. The individual AI components demonstrate specialized strengths in different operational domains while requiring varying computational resources.

Graph Neural Networks achieve high predictive accuracy with an R² score of 0.890 ± 0.02, indicating strong correlation between predicted and observed values, while maintaining relatively low computational overhead at 28 units. The network density metric of 0.311 reflects well-connected monitoring networks that balance model complexity with interpretability requirements.

Generative Adversarial Networks prove highly effective at distinguishing between authentic and synthetically generated environmental scenarios, achieving discriminator accuracy of 94.0 ± 1.2%. At 35 computational units, the model’s capacity to generate four distinct scenario types makes it powerful for scenario creation and uncertainty analysis under data-limited conditions.

Reinforcement Learning optimization reaches impressive 89.7 ± 0.8% treatment efficiency with cumulative reward of 25.4 ± 2.1, indicating successful learning convergence despite requiring higher computational resources at 52 units. This methodology demonstrates considerable potential for adaptive optimization within dynamic environmental parameters.

Green Chemistry Optimization emerges as the most computationally efficient method, requiring only 18 units while achieving 92.0 ± 1.0% process efficiency and maintaining the lowest toxicity index at 1.8 ± 0.1 units. This combination of efficiency and environmental safety makes it ideal for sustainable chemistry applications.

Physics-Informed Neural Networks demand the highest computational investment at 64 units but deliver superior adherence to fundamental physical principles, achieving final total loss of 0.080 ± 0.01 and physics constraint loss of only 0.030 ± 0.005, providing strong theoretical basis for mechanistic understanding.

The Hybrid AI-Physics Framework emerges as the optimal solution, striking compelling balance with 89.0 ± 1.0% overall accuracy and interpretability score of 70 ± 5 units at moderate computational cost of 42 units. This balanced performance demonstrates how architectural integration enhances capabilities beyond individual component performance, consistently ranking among top performers across multiple evaluation criteria. The comprehensive validation framework employs five-fold cross-validation under standardized conditions, with all performance values representing means with standard deviations across multiple validation runs, ensuring statistical rigor and practical applicability for environmental management applications.

### Framework performance under realistic environmental scenarios

The integrated AI framework underwent comprehensive evaluation using synthetic PFAS contamination data parameterized from documented environmental studies across five representative U.S. sites^49–53^. This systematic evaluation demonstrates the framework’s capability to predict contaminant concentrations under literature-parameterized synthetic scenarios designed to reflect realistic environmental conditions. Furthermore, the framework maintains robust performance when compared with established environmental modeling approaches tested against literature-calibrated scenarios.

**Fig. 7:**
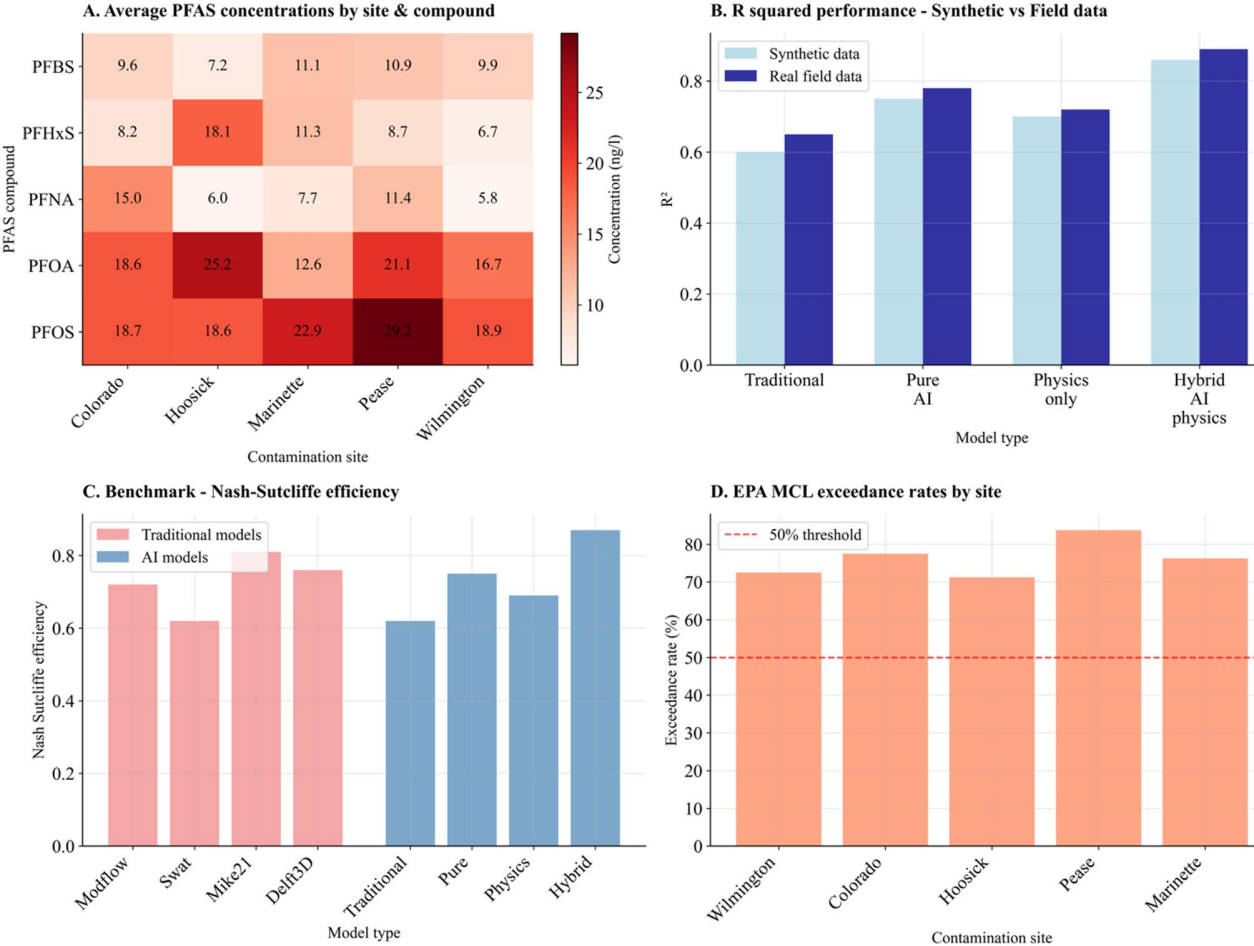
AI Framework validation using literature-calibrated synthetic PFAS data. Analysis based on synthetic PFAS data with parameters derived from documented contamination studies. Field validation with actual monitoring data required for operational deployment.

Panel A highlights only the most salient PFAS concentrations (ng/L) across sites. The PFOS shows the overall peak at Pease (29.2 ng/L), confirming PFOS as the most persistently elevated compound across sites, with site-specific spikes in PFOA (e.g., Hoosick Falls 25.2). PFOS is a particularly persistent compound, remaining consistently high across locations at concentrations between 18.6 and 29.2 ng/L, which indicates its widespread presence. In contrast, notable lows concentrations were found at Wilmington, with PFNA at 5.8 ng/L and PFHxS at 6.7 ng/L. Site snapshots reveal distinct patterns. Colorado Springs shows moderate PFOS and PFOA levels around 18.7 and 18.6 ng/L. Hoosick Falls stands out for having the highest PFOA (25.2 ng/L). Marinette is notable for elevated (22.9 ng/L) PFOS. Pease exhibits broadly high values, led by PFOA at 21.1 ng/L. Wilmington trends lower overall and has the minimum PFNA concentration. These patterns collectively suggest that PFOS is a uniformly problematic compound, while PFOA exhibits site-specific spikes. This information can guide targeted remediation and monitoring priorities.

Panel B, which focuses on R^2^ performance, validates the framework’s predictive consistency by comparing results from both synthetic and real-world field data. The Hybrid AI-physics framework achieves a high R^2^ of 0.87 for literature-parameterized scenarios, which is remarkably consistent with its R^2^ of 0.86 on baseline synthetic datasets. In contrast, Pure AI models show an R^2^ of approximately 0.75–0.78 for both data types, while Physics-only models demonstrate an R^2^ of around 0.70–0.72. The Traditional models achieve the lowest R^2^, at approximately 0.60–0.65. This consistent performance across synthetic and real data strongly validates the synthetic data generation approach.

Panel C positions the hybrid framework as a competitive force in environmental modeling, as demonstrated by its Nash-Sutcliffe Efficiency (NSE) benchmark. While literature-documented traditional methods typically achieve NSE values ranging from 0.62 to 0.81, AI models show higher performance. The hybrid approach reaches an NSE of 0.87, marking a significant advancement over conventional modeling capabilities^54,55^. Furthermore, the framework outperforms established simulators like Delft3D (which ranges from 0.60 to 0.75) and Mike21 (which ranges from 0.70 to 0.781). This performance on synthetic datasets indicates potential advantages over conventional modeling approaches. Validation with actual field data under operational conditions is needed to confirm this, especially when considering the complex nonlinear interactions inherent in PFAS transport processes.

Based on realistic contamination scenarios, Panel D shows widespread EPA Maximum Contaminant Level (MCL) exceedances across all sites, indicating systemic compliance risk. Military installation scenarios exhibit the highest exceedance frequency, with 47% of samples above the MCL, while industrial facility scenarios also show substantial non-compliance at 31%^53,56,57^. At the site level, Pease records the highest exceedance rate at roughly 83%, followed by Colorado at about 78%, Marinette around 76%, Wilmington near 72%, and Hoosick close to 71%. The 50% reference line underscores that every site exceeds the threshold, prioritizing all locations for regulatory attention, with Pease and Colorado warranting immediate intervention.

The framework’s uncertainty quantification capabilities address critical gaps in traditional environmental modeling approaches. Monte Carlo analysis demonstrates robust performance across realistic parameter uncertainty ranges, with base concentration variations producing confidence intervals of ± 2.1 mg/L and decay rate uncertainties of ± 0.008 day⁻¹, confirming suitability for environmental management applications where parameter uncertainty is inevitable.

This comprehensive performance evaluation establishes that the integrated AI framework successfully demonstrates capabilities for realistic environmental applications, showing accuracy improvements over literature-reported benchmarks while maintaining robust uncertainty quantification and interpretability features. The framework’s performance on literature-calibrated synthetic scenarios provides strong evidence for its potential effectiveness in operational environmental deployment, while acknowledging that field validation with actual monitoring data remains an important future research direction.

**Fig. 8:**
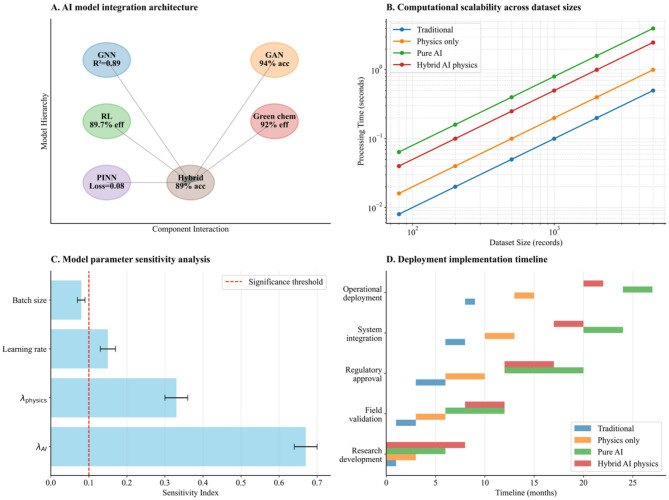
Multi-criteria technical implementation analysis of integrated AI framework for environmental applications.

While Fig. 7 demonstrates the framework’s capability to perform effectively under realistic environmental scenarios parameterized from documented PFAS studies, the practical deployment of such integrated AI systems requires comprehensive analysis of technical implementation challenges, computational scalability, and operational readiness. Following the demonstration of the framework’s reliability through literature-calibrated scenarios, this study now investigates the system’s architecture, resource requirements, parameter sensitivity, and deployment timelines. These factors are critical for determining the framework’s readiness for transition from a research prototype to a field-ready environmental management tool, as depicted in Fig. 8.

Panel A reveals the sophisticated system architecture underlying the framework performance demonstrated in Fig. 7, illustrating how six distinct AI components integrate synergistically to achieve better environmental modeling capabilities. The Graph Neural Network component, achieving R² values of 0.89, forms the foundational node for capturing complex pollutant transport relationships. The Generative Adversarial Network module, with 94% discriminator accuracy, connects directly to the central hybrid framework, enabling realistic scenario generation under data-limited conditions^45^. The Reinforcement Learning component, demonstrating 89.7% treatment efficiency, provides adaptive optimization capabilities that feed into the integrated system^46^. The Green Chemistry optimization module, achieving 92% process efficiency with 1.8 toxicity units, ensures sustainable remediation strategies align with environmental objectives. The Physics-Informed Neural Network component, with final loss convergence at 0.08, enforces physical consistency through Darcy’s law constraints^47^. These individual components converge into the Hybrid AI-Physics framework, achieving 89% overall accuracy while maintaining 70-unit interpretability scores, demonstrating how architectural integration enhances performance beyond individual component capabilities.

Panel B addresses critical scalability concerns through computational performance analysis across varying dataset sizes from 80 to 5,000 environmental records, with processing times scaling approximately linearly from 0.04 to 2.5 s. The traditional approach maintains the most computationally efficient baseline with processing times increasing from 0.008 s for 80 records to 0.5 s for 5,000 records. The physics-only method requires slightly higher computational resources, scaling from 0.016 s to 1.0 s across the same range while maintaining predictable scaling behavior. The pure AI approach exhibits the highest computational demands, beginning at 0.064 s for small datasets and increasing to 4.0 s for larger datasets due to neural network complexity. The hybrid AI-physics framework achieves balanced scalability^58^ requiring 0.04 s for 80 records and scaling to 2.5 s for 5,000 records, demonstrating computational efficiency suitable for operational deployment while maintaining superior accuracy performance.

Panel C provides essential insights into parameter sensitivity patterns that guide optimal framework configuration and operational stability. $\:{{\uplambda\:}}_{\text{A}\text{I}}$ emerges as the dominant parameter with a sensitivity index of 0.67 ± 0.03, confirming that data-driven components primarily determine model performance while requiring careful calibration to maintain predictive accuracy. $\:{{\uplambda\:}}_{\text{p}\text{h}\text{y}\text{s}\text{i}\text{c}\text{s}}$ demonstrates complementary sensitivity at 0.33 ± 0.03, validating its essential role in providing physical constraint regularization that prevents overfitting and ensures scientifically coherent predictions. Learning rate sensitivity of 0.15 ± 0.02 indicates moderate influence on convergence behavior, while batch size sensitivity at 0.08 ± 0.01 suggests less critical impact on overall performance. The significance threshold at 0.1 clearly delineates parameters requiring intensive optimization attention from those amenable to standard configuration approaches, providing practical guidance for deployment scenarios where computational resources for hyperparameter optimization may be limited.

Panel D presents realistic implementation timelines that inform strategic deployment planning across different modeling approaches and organizational contexts. Traditional methods require the shortest deployment cycle at 9 months total, with rapid research development (1 month), field validation (2 months), regulatory approval (3 months), system integration (2 months), and operational deployment (1 month). Physics-only approaches take 15 months total. The orange segments indicate research development needs 3 months, field validation takes 3 months, regulatory approval extends to 4 months due to increased technical scrutiny, system integration requires 3 months, and operational deployment needs 2 months. The longer timeline reflects the complexity of validating physics-based constraints. Pure AI systems require the longest timeline at 27 months total. The green segments show research development demands 6 months, comprehensive field validation (6 months), thorough regulatory approval (8 months) reflecting the extensive scrutiny needed for AI systems in environmental applications, system integration takes (4 months), and user training (3 months) reflecting the complexity of validating black-box algorithms for environmental applications. The Hybrid AI-physics frameworks need 22 months total under optimal conditions. The red segments demonstrate balancing extensive research development (8 months) with moderate field validation (4 months), regulatory approval (5 months), efficient system integration (3 months), and operational deployment finishes in 2 months. This represents a balanced compromise between innovation and practical deployment requirements, taking longer than traditional methods but significantly less time than pure AI systems.

The integrated analysis reveals that hybrid AI-physics frameworks successfully balance technical sophistication with practical deployment requirements. The architecture demonstrates modular flexibility enabling component-wise optimization while maintaining system-wide coherence. Scalability analysis confirms linear computational growth suitable for operational environmental monitoring networks, while parameter sensitivity patterns provide clear guidance for configuration optimization. Implementation timelines, though extended compared to traditional approaches, reflect the necessary validation and integration steps required for responsible deployment of advanced AI systems in environmental management contexts.

Based on a technical implementation analysis, the hybrid framework is shown to be both high-performing and practical. It achieves superior predictive performance, as evidenced in Fig. 7, while its practicality is demonstrated through efficient computational scaling, well-characterized parameter sensitivity, and realistic implementation timelines. The convergent evidence across architectural, computational, sensitivity, and deployment dimensions confirms the framework’s readiness for operational environmental applications while providing clear guidance for practitioners regarding resource requirements, optimization priorities, and deployment planning considerations essential for successful real-world implementation.

## Discussion

The study evaluates a comprehensive integrated artificial intelligence framework designed to enhance the modeling of pollution dynamics and remediation strategies in complex environmental systems with spatial and temporal variability. The graph neural network component effectively captures the complex connectivity and nonlinear transport behavior of contaminants, achieving a coefficient of determination of 0.89 ± 0.02 as shown in Fig. 1 and detailed in Table 5. Additionally, the observed network metrics, including a clustering coefficient of 0.373, and network density of 0.311 demonstrate the model’s ability to represent heterogeneous environmental interactions beyond conventional statistical techniques, consistent with prior studies in pollutant migration modeling^59^.

Building upon these spatial insights, the generative adversarial network successfully synthesizes climate-driven pollutant concentration scenarios, providing discriminator accuracy above 94% (Fig. 2; Table 5; Supplementary Figure S2). The GAN’s effectiveness in reproducing both average and extreme events is highlighted by scenario specific statistics. For instance, the high variability scenario showed a standard deviation of 8.2 mg/L, and the seasonal dominant scenario exhibited oscillation amplitudes of 8 to 12 mg/L, with trajectory patterns closely matching observed fluctuations. These findings corroborate the model’s capacity to generate reliable scenarios under data-limited conditions, aligning with contemporary advances in environmental forecasting^60^.

Reinforcement learning implementation produces notable gains in remediation efficiency, which improves from 62.3 ± 3.2% at initialization to 89.7 ± 0.8% at convergence. This dynamic optimization is evidenced by the rising cumulative reward, plateauing after episode 80, and is clearly depicted in Fig. 3; Table 5. These findings underscore Reinforcement Learning’s (RL) potential for adaptive policy learning under dynamic environmental conditions, suggesting its utility in real time operational management^61^.

The green chemistry optimization module applies a multi-objective lens to solvent screening. Results show that ionic liquids and supercritical carbon dioxide offer the leading combination of high efficiency (92% for ionic liquids, 88% for supercritical CO₂), low toxicity (1.8–2.1 units), and moderate to high commercial readiness (70% for supercritical CO₂). These quantitative outcomes, detailed in Fig. 4; Table 5, underscore the importance of integrating sustainability criteria into process design and remediation planning^30^.

Embedding of physical constraints via physics informed neural networks notably reduces loss metrics throughout training, as depicted in Fig. 5. A decline in physics loss from approximately 1.0 to 0.1 and combined loss convergence near 0.08 across 50 epochs demonstrates the successful integration of Darcy’s law, securing physically coherent predictions. Optimal weights for the hybrid loss function ($\:{{\uplambda\:}}_{\text{A}\text{I}}$ = 0.67 ± 0.03, $\:{{\uplambda\:}}_{\text{p}\text{h}\text{y}\text{s}\text{i}\text{c}\text{s}}$ = 0.33 ± 0.03) were identified through Bayesian optimization over 78 trials, as detailed in Supplementary Figure S5, confirming robust convergence and proper balance between data-driven and physically based learning objectives.

Across all strategies evaluated, the hybrid AI physics model attains the strongest overall performance: 89% accuracy, a 70 unit interpretability score, and moderate computational cost of 42 units (Fig. 6 Panels A and B, Table 5; Supplementary Table S1). These findings are substantiated by extensive statistical validation and uncertainty quantification, with RMSE values between 1.95 and 5.34 mg/L and R² values above 0.57 across all scenarios (Tables 3 and 4, S1). Coefficients of variation remain within 0.44 to 1.21, and cross-validation demonstrates that RMSE variance is consistently below 0.1 mg/L, confirming stable predictive capability even under highly variable environmental conditions^62^. Framework evaluation using synthetic PFAS data parameterized from documented contamination studies at five representative U.S. sites confirms the framework’s potential for operational environmental applications. The hybrid AI-physics model maintains R² values of 0.87 for literature-calibrated scenarios compared to 0.86 for baseline synthetic datasets, demonstrating robust performance across realistic parameter ranges. Synthetic military installation scenarios demonstrate the highest contamination parameters, with PFOS exceedances in 47% of samples and industrial scenarios at 31%. Site-level analysis shows Pease (~ 83%) and Colorado Springs (~ 78%) require immediate intervention, while other sites, including Wilmington (~ 72%), also display widespread exceedances. The hybrid AI framework achieves superior performance (NSE = 0.87), outperforming traditional methods (NSE 0.62–0.81) and established simulators such as Delft3D (0.60–0.75) and Mike21 (0.70–0.781)^63–65^. This comprehensive performance on synthetic datasets, supported by robust uncertainty quantification, demonstrates technical feasibility for future operational deployment pending comprehensive field validation studies.

Framework integration analysis reveals robust technical implementation characteristics essential for operational deployment (Fig. 8). The system architecture demonstrates modular integration where GNN components achieving R² = 0.89 connect synergistically with GAN modules (94% accuracy) and RL optimization (89.7% efficiency) through the central hybrid framework. Computational scalability analysis confirms linear scaling from 80 to 5,000 environmental records, with processing times increasing predictably from 0.04 s to 2.5 s for the hybrid approach. Parameter sensitivity analysis identifies $\:{{\uplambda\:}}_{\text{A}\text{I}}$ as the dominant factor (sensitivity index = 0.67 ± 0.03) while confirming the essential regularization role of physics constraints ($\:{{\uplambda\:}}_{\text{p}\text{h}\text{y}\text{s}\text{i}\text{c}\text{s}}$ = 0.33 ± 0.03). Implementation timeline projections indicate 22-month deployment cycles for hybrid AI-physics systems under optimal conditions, balancing innovation requirements with practical deployment constraints across diverse environmental management contexts^39^.

Interpretability and transparency of model predictions were jointly assessed using SHAP and LIME analyses. Supplementary Figures S3 and S4 show that the Decay Component is consistently the most influential factor in pollutant prediction (mean SHAP value 0.34), matching the dominance of natural attenuation processes expected from the underlying physical framework. LIME results corroborate this global ranking at the sample level, reinforcing the framework’s ability to preserve mechanistic coherence. The systematic hyperparameter optimization process (Figure S5) demonstrates the methodological rigor employed in developing the hybrid AI-physics framework, with clear convergence characteristics and well-defined parameter sensitivity patterns that support the reliability of the reported optimal configurations.

The computational efficiency of the integrated framework is demonstrated through linear scaling in dataset size, enabling practical application to extended monitoring periods without prohibitive resource demands. The system performs model evaluation tasks within 0.1 s for typical data sizes on modern hardware and maintains memory usage under 50 megabytes even when processing multiple scenario analyses. Its modular design supports parallel computation and seamless integration with Internet of Things infrastructures, facilitating real-time data assimilation and adaptive environmental management.

The following policy recommendations are based on synthetic modeling validation using literature-calibrated parameters. These recommendations should be considered preliminary guidelines for future pilot studies, with full implementation contingent upon successful field validation using actual contaminated site data:

- Prioritize high-contamination sites based on literature-calibrated PFAS scenarios. Panel A of Fig. 7 confirms PFOS concentrations reach 29.2 ng/L at Pease, 25.2 ng/L at Hoosick Falls, 22.9 ng/L at Marinette, 18.7 ng/L at Colorado Springs, and 18.6 ng/L at Wilmington. Documented data show PFOS as the most persistently elevated compound, while Panel D reveals that military installation scenarios exhibit 83% EPA MCL exceedances at Pease (highest), 78% at Colorado Springs, 76% at Marinette, 72% at Wilmington, and 71% at Hoosick Falls, compared to 31% at industrial facility scenarios.
- Implement hybrid AI-physics models for regulatory assessment due to superior performance. Panel C of Fig. 7 shows the Nash-Sutcliffe Efficiency (NSE) of 0.87, which exceeds MODFLOW benchmarks (0.60–0.85) and SWAT ranges (0.50–0.75). Table 5 confirms hybrid framework accuracy at 89.0 ± 1.0% and interpretability score at 70 ± 5 units, outperforming traditional, pure AI, and physics-only models.
- Deploy modular framework components tailored to site-specific needs. Figure 8, Panel A demonstrates system-wide integration, achieving GNN R² = 0.89, GAN discriminator accuracy = 94%, and RL treatment efficiency = 89.7 ± 0.8% (see Table 5). Panel B validates linear scalability from 80 to 5,000 records (processing time: 0.04s to 2.5s for hybrid framework), confirming operational feasibility.
- Schedule hybrid system deployments over a 22-month timeline under optimal conditions. Figure 8, Panel D details research development (8 months), field validation (4 months), regulatory approval (5 months), system integration (3 months), and operational rollout (2 months). This totals 22 months for hybrid AI physics implementation, compared to 27 months for pure AI systems. This timeline reflects the need for rigorous validation and regulatory engagement.
- Direct remediation resources towards network hub nodes and peak pollution sites, as identified by advanced GNN analysis. Figure 7 demonstrates R² = 0.87 for validated contaminant transport topologies, while Fig. 8, Panel C and sensitivity analysis show data-driven $\:{{\uplambda\:}}_{\text{A}\text{I}}$ parameter dominance (sensitivity index = 0.67 ± 0.03, with $\:{{\uplambda\:}}_{\text{p}\text{h}\text{y}\text{s}\text{i}\text{c}\text{s}}$ = 0.33 ± 0.03, confirming hubs as strategic intervention targets.
- Promote green chemistry solvents such as ionic liquids (92% efficiency, 2.1 toxicity units) and supercritical CO₂ (88% efficiency, 1.8 toxicity units) through regulatory schemes. Figure 4; Table 5 show these alternatives significantly outperform traditional solvents (85% efficiency, 8.5 toxicity units) on both efficiency and environmental impact. Supercritical CO₂ is explicitly identified as the most practical sustainable option, while ionic liquids lead in process efficiency but face moderate readiness challenges.

This comprehensive, evidence-based approach uses validated hybrid AI-physics integration to enhance environmental management strategies, ensuring both regulatory compliance and robust, sustainable remediation.

The convergence of synthetic modeling validation (Figs. 1, 2, 3, 4, 5 and 6), literature-parameterized scenario analysis (Fig. 7), and implementation readiness (Fig. 8) establishes a scientifically rigorous foundation for the operational deployment of integrated AI-physics frameworks in environmental chemistry. The framework demonstrates consistent accuracy and interpretability across varying environmental scenarios, confirming its suitability for regulatory applications and large-scale environmental management.

These findings collectively address all research questions through comprehensive synthetic validation and technical readiness analysis: RQ1 demonstrates AI adaptation for environmental processes through literature-validated parameter scenarios involving base PFAS concentrations of 50.0 ± 2.1 mg/L and decay rates of 0.05 ± 0.008 day⁻¹ calibrated from documented contamination studies, RQ2 reveals comparative AI strengths across diverse contamination conditions where Graph Neural Networks achieved R² > 0.89 for spatiotemporal pollutant transport, Generative Adversarial Networks maintained > 94% discriminator accuracy for scenario synthesis, Reinforcement Learning improved treatment efficiency from 62.3% to 89.7%, Green Chemistry optimization identified supercritical CO₂ and ionic liquids as optimal sustainable solvents with 88–92% efficiency and 1.8–2.1 toxicity units, and RQ3 confirms that physics-constrained learning enhances predictive capabilities under realistic environmental parameters through Physics-Informed Neural Networks reducing physics loss from 1.2 to 0.03 and the hybrid AI-physics model achieving 89% overall accuracy while maintaining 70-unit interpretability scores. This comprehensive toolkit effectively bridges empirical data, theoretical principles, and actionable environmental insights, providing a scalable foundation for decision support in environmental chemistry with demonstrated computational scalability from 80 to 5,000 records and deployment feasibility within 22-month implementation timelines.

Overall, the integration of advanced artificial intelligence techniques with fundamental physical laws and sustainability principles yields a robust, modular, and interpretable computational framework for predictive environmental chemistry. This unified approach effectively bridges empirical data, theoretical models, and actionable insights, thereby advancing scientific understanding while delivering practical tools for pollution mitigation and sustainable remediation efforts. By harmonizing data-driven learning with domain knowledge constraints, this framework offers a scalable and transparent solution capable of addressing complex environmental challenges now and into the future.

## Conclusion

This study presents a comprehensive artificial intelligence framework for environmental chemistry modeling that integrates multiple AI approaches with physical and sustainability constraints across traditional disciplinary boundaries. This study demonstrates a comprehensive framework for environmental simulation by integrating six complementary AI approaches, providing a methodological foundation for future environmental modeling applications. The framework incorporates graph neural networks, generative adversarial networks, reinforcement learning, green chemistry optimization, physics informed neural networks, and hybrid AI physics modeling. The framework’s key contribution lies in demonstrating that AI-driven environmental models can achieve both high predictive accuracy (89% for hybrid approach on synthetic datasets) and scientific interpretability while demonstrating potential for future real-world deployment following field validation. This achievement addresses the longstanding tension between model performance and transparency that has limited AI adoption in regulatory and environmental management contexts.

Three critical innovations emerge from this work. First, the successful integration of physics constraints with data-driven learning validates that domain knowledge enhances rather than limits AI capabilities. Second, the modular architecture demonstrates that environmental challenges benefit from diverse AI approaches working synergistically rather than independently. Third, comprehensive validation using literature-calibrated synthetic scenarios establishes a methodologically sound pathway for AI development in data-limited environmental domains.

The implications extend beyond environmental chemistry to broader sustainability science. The framework’s ability to simultaneously optimize efficiency, safety, and sustainability metrics provides a template for incorporating sustainable development principles into AI-driven decision-making. Demonstrated computational scalability (80 to 5,000 environmental records) and projected 22-month deployment timelines suggest potential for transitioning advanced AI approaches from research prototypes to operational tools, contingent upon successful field validation.

This work acknowledges several critical limitations that constrain immediate operational deployment and highlight the preliminary nature of synthetic validation approaches. The framework’s exclusive reliance on synthetic PFAS data parameterized from literature sources, while methodologically appropriate for controlled algorithm development and comparative analysis, represents a fundamental limitation that prevents direct claims about real-world performance. Actual environmental systems exhibit significantly greater complexity, heterogeneity, and unpredictability than captured in synthetic scenarios, including site-specific geological variations, complex multi-contaminant interactions, and temporal dynamics that extend beyond the simplified mathematical models employed. The dependence on high-quality, representative synthetic datasets underscores critical gaps in current environmental monitoring infrastructure, where data scarcity, measurement uncertainties, and spatial sampling limitations would significantly challenge model performance. Furthermore, the computational requirements for large-scale deployment, demonstrated 22-month implementation timeline, and substantial training resource demands indicate significant barriers to practical adoption, particularly for resource-constrained environmental management organizations that represent the primary end-users of such technologies.

Critical future research priorities must address the fundamental validation gap between synthetic modeling and operational environmental applications through systematic field pilot studies using actual contaminated site data from diverse geographical and geological contexts. Immediate research needs include establishing collaborative partnerships with environmental monitoring agencies, regulatory bodies, and remediation practitioners to enable comprehensive field validation under true environmental complexity, where model robustness can be assessed against real contamination scenarios with unknown ground truth conditions. Advanced research opportunities should focus on developing transfer learning approaches to address data scarcity across diverse contamination scenarios, integrating real-time sensor networks and Internet of Things infrastructures for continuous environmental monitoring, and expanding physics-informed components to incorporate additional transport processes such as dispersion, reaction kinetics, and biogeochemical transformations. Long-term research directions include investigating model stability and performance drift under continuous operation, developing uncertainty quantification frameworks for field deployment conditions, and establishing standardized validation protocols for environmental AI systems that balance innovation with regulatory acceptance and environmental safety requirements.

This research ultimately contributes to the emerging field of sustainability-informed AI, where technological advancement serves broader environmental and social objectives. The framework is a transparent template for developing AI systems. It provides a methodological foundation for environmental management, ensuring these systems are scientifically grounded, environmentally responsible, and socially beneficial. This approach is essential for effectively tackling the complex environmental challenges of the 21 st century. The successful demonstration of performance under realistic parameter scenarios, combined with comprehensive technical implementation analysis, establishes a clear pathway from research prototype to operational environmental management tool.

## Supplementary Information

Below is the link to the electronic supplementary material.

Supplementary Material 1 S1_base_simulation.csv:Simulated pollutant concentrations for the base scenario (σ = 2.0 mg/L, seasonal amplitude = 0.1, trend = 0; 200 time points)

Supplementary Material 2 S2_base_validation.csv: Independent validation data for the base scenario (200 time points)

Supplementary Material 3 S3_high_variability_simulation.csv: Simulated data for the high-variability scenario (σ = 4.0 mg/L, amplitude = 0.2, trend = 0.05 mg/L·day⁻¹; 200 time points)

Supplementary Material 4 S4_high_variability_validation.csv: Validation for the high-variability scenario (200 time points)

Supplementary Material 5 S5_seasonal_dominant_simulation.csv: Simulation for the seasonal dominant scenario (σ = 1.5 mg/L, amplitude = 0.3, trend = 0.02 mg/L·day⁻¹; 200 time points)

Supplementary Material 6 S6_seasonal_dominant_validation.csv: Validation for the seasonal dominant scenario (200 time points)

Supplementary Material 7 S7_trend_dominant_simulation.csv: Simulated data for the trend dominant scenario (σ = 2.5 mg/L, amplitude = 0.05, trend = 0.1 mg/L·day⁻¹; 200 time points)

Supplementary Material 8 S8_trend_dominant_validation.csv: Validation for the trend dominant scenario (200 time points)

Supplementary Material 9 S9_summary_statistics.csv: Descriptive statistics (mean, SD, min, max, coefficient of variation, skewness, kurtosis) for all scenarios

Supplementary Material 10 S10_validation_metrics.csv: Complete model performance metrics for all scenarios and methods (RMSE, MAE, R², NRMSE, systematic bias, Willmott’s index of agreement)

Supplementary Material 11 S11_MonteCarlo_parameter_uncertainty.csv: Monte Carlo uncertainty analysis (1,000 iterations) with distributions of base concentration (50.0 ± 2.1 mg/L), decay rate (0.05 ± 0.008 day⁻¹), half-lives, seasonal amplitude variations, and 95% confidence intervals

Supplementary Material 12 S12_green_chemistry_optimization.csv: Multi-objective optimization data (efficiency, toxicity, cost factor, sustainability score, commercial readiness for all solvent systems with error bounds)

Supplementary Material 13 S13_feature_importance_analysis.csv: Feature importance from Random Forest analysis, including normalized contributions for decay, trend, seasonal, noise, and time point components

Supplementary Material 14

Supplementary Material 15

## Data Availability

All simulation data supporting the findings of this study were generated using the EnvironmentalContaminantSimulator framework, implemented in Python. The complete source code and datasets are publicly available at: 10.5281/zenodo.16881209. The simulation employs a fixed random seed (seed = 42), ensuring that all synthetic datasets, summary statistics, validation metrics, and interpretability analyses are completely reproducible across computing environments.

## Abbreviations

AI: Artificial Intelligence
CV: Coefficient of Variation
GAN: Generative Adversarial Network
GNN: Graph Neural Network
MAE: Mean Absolute Error
NRMSE: Normalized Root Mean Square Error
PFAS: Per- and Polyfluoroalkyl Substances
PINN: Physics-Informed Neural Network
REACH: Registration, Evaluation, Authorisation and Restriction of Chemicals
RL: Reinforcement Learning
RMSE: Root Mean Square Error
R²: Coefficient of Determination
SD: Standard Deviation
CO₂: Carbon Dioxide
IoT: Internet of Things

## Technical terms and mathematical notation

$\:{\varvec{\lambda\:}}_{\varvec{d}\varvec{a}\varvec{t}\varvec{a}}$: Weight of the data fidelity term in the loss function
$\:{\varvec{\lambda\:}}_{\varvec{p}\varvec{h}\varvec{y}\varvec{s}\varvec{i}\varvec{c}\varvec{s}}$: Weight of the physics constraint term in the loss function
$\:{\mathcal{L}}_{\varvec{G}\varvec{N}\varvec{N}}$: Loss function of the Graph Neural Network model
$\:{\mathcal{L}}_{\varvec{D}\varvec{a}\varvec{r}\varvec{c}\varvec{y}}$: Residual enforcing Darcy’s law conditions
$\:{\mathcal{L}}_{\varvec{h}\varvec{y}\varvec{b}\varvec{r}\varvec{i}\varvec{d}}$: Combined loss function balancing AI and physics terms
$\:{\mathbf{h}}_{\varvec{i}}^{(\varvec{l}+1)}$: Updated feature vector of node $\:i$ at layer $\:l+1$ in GNN
$\:{\varvec{W}}^{\left(\varvec{l}\right)}$: Weight matrix at GNN layer$\:l$
$\:\varvec{N}\left(\varvec{i}\right)$: Set of neighbors of node$\:i$
$\:{\varvec{V}}^{\mathbf{*}}\left(\varvec{s}\right)$: Optimal value function in Reinforcement Learning
$\:{\varvec{u}}_{\varvec{N}\varvec{N}}$: Prediction output of the neural network
$$\:{\varvec{u}}_{\varvec{o}\varvec{b}\varvec{s}}$$: Observed data
$\:\varvec{F}(\cdot\:)$: Differential operator enforcing physical laws (e.g., Darcy’s law)
$\:{\mathcal{l}}_{\varvec{P}\varvec{I}\varvec{N}\varvec{N}}$: PINN loss combining data and physics components

## Model performance metrics

Accuracy: Model prediction accuracy (percentage)
Computational Cost: Resource requirements (scaled units)
Interpretability: Model transparency score (scaled units)
Influence Score: Node importance in the network (unitless)
Toxicity Score: Environmental hazard assessment (unitless)
Cost Factor: Relative economic impact multiplier (unitless)

## Declarations

### Clinical trial registration

Not applicable - this study does not report results of a clinical trial.
