# Supplementary material for: Unified artificial intelligence framework for modeling pollution dynamics and sustainable remediation in environmental chemistry
Source: Sci Rep. 2025 Oct 16;15:36196. doi: 10.1038/s41598-025-20083-w (PMC12533017; doi:10.1038/s41598-025-20083-w)
Supplement: Supplementary file 15 — Supplementary Material 15 [file 41598_2025_20083_MOESM15_ESM.docx]

Supplementary Information for:

**Unified Artificial Intelligence Framework for Modeling Pollution Dynamics and Sustainable Remediation in Environmental Chemistry**

Author: **Mohammad Fazle Rabbi^a,^***

^a,^* Coordination and Research Centre for Social Sciences, Faculty of Economics and Business, University of Debrecen, Böszörményi út 138, 4032 Debrecen, Hungary.

*Correspondence: [drrabbikhan@gmail.com](mailto:drrabbikhan@gmail.com); or [rabbi.mohammad@econ.unideb.hu](mailto:rabbi.mohammad@econ.unideb.hu)

**Table of Contents**

**S1.** Residual diagnostics (Supplementary Figure S1) .......... p.2

**S2.** Monte Carlo uncertainty (Supplementary Figure S2) ...... p.3

**S3.** SHAP feature importance (Supplementary Figure S3) ...... p.5

**S4.** LIME local explanation (Supplementary Figure S4) ........ p.7

**S5.** Fivefold cross-validation results (Supplementary Table S1) .... p.10

**S6.** Monte Carlo parameter summary (Supplementary Table S2) . p.11


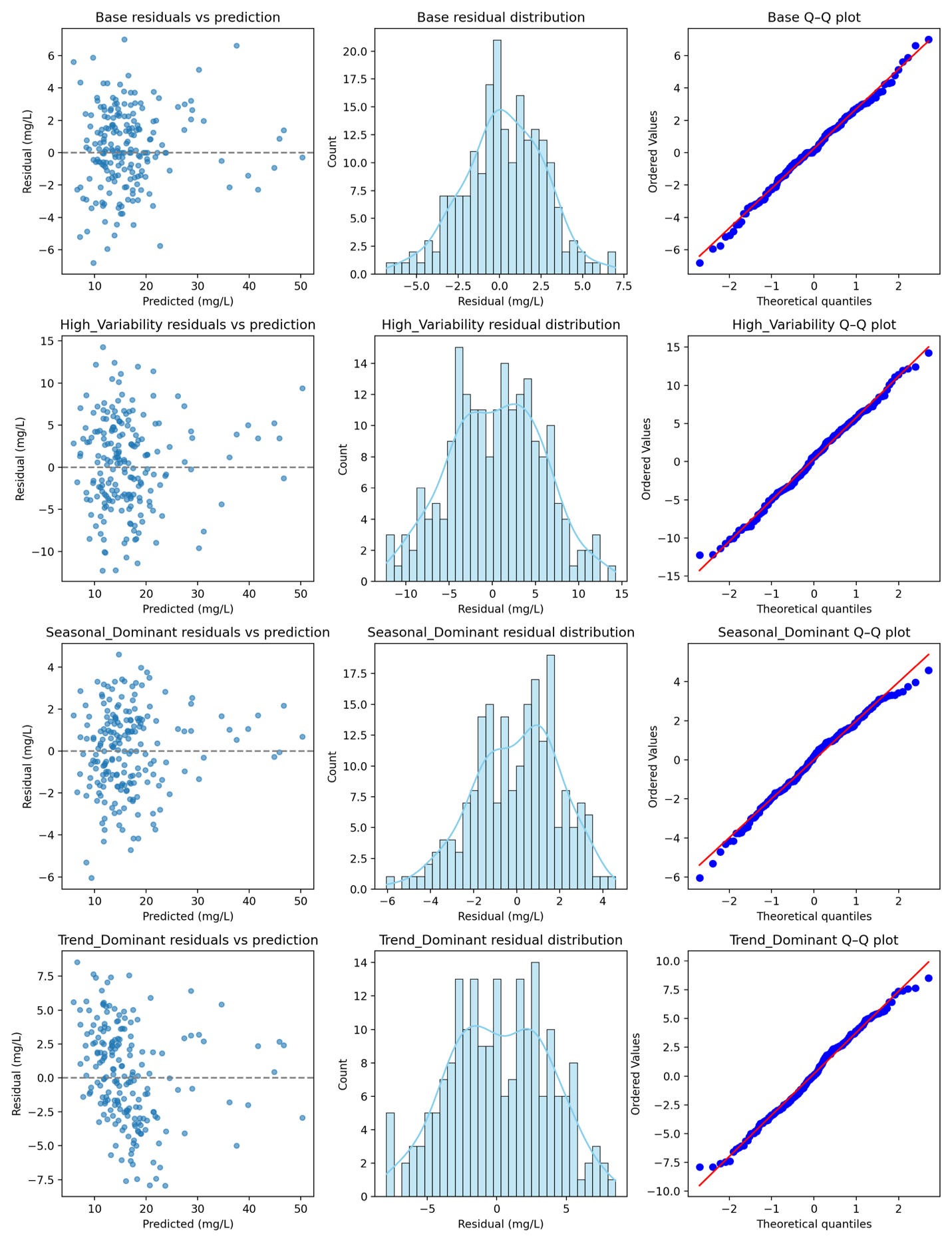


**Supplementary Figure S1.** Residual diagnostics for pollutant concentration predictions across environmental scenarios

Supplementary Figure S1 presents a comprehensive set of residual diagnostics for the four environmental scenarios evaluated in this study (base, high variability, seasonal dominant, and trend dominant). The first column displays scatter plots of residuals versus predicted concentrations, showing that residuals are symmetrically distributed around zero with no apparent heteroscedastic pattern, indicating unbiased model predictions across the range of concentrations. The second column provides histograms overlaid with kernel density estimates for each scenario, which reveal approximately normal distributions of residuals centered near zero and similar spread, confirming that the magnitude of errors remains consistent across scenarios. The third column features Q–Q plots comparing ordered residuals to theoretical normal quantiles, demonstrating close alignment with the 45° reference line and only slight deviations in the distribution tails, supporting the assumption of normality in error structure. Collectively, these diagnostic plots validate that the model’s residuals are approximately Gaussian, unbiased, and homoscedastic, thereby reinforcing the robustness and reliability of the simulation framework used in this study.


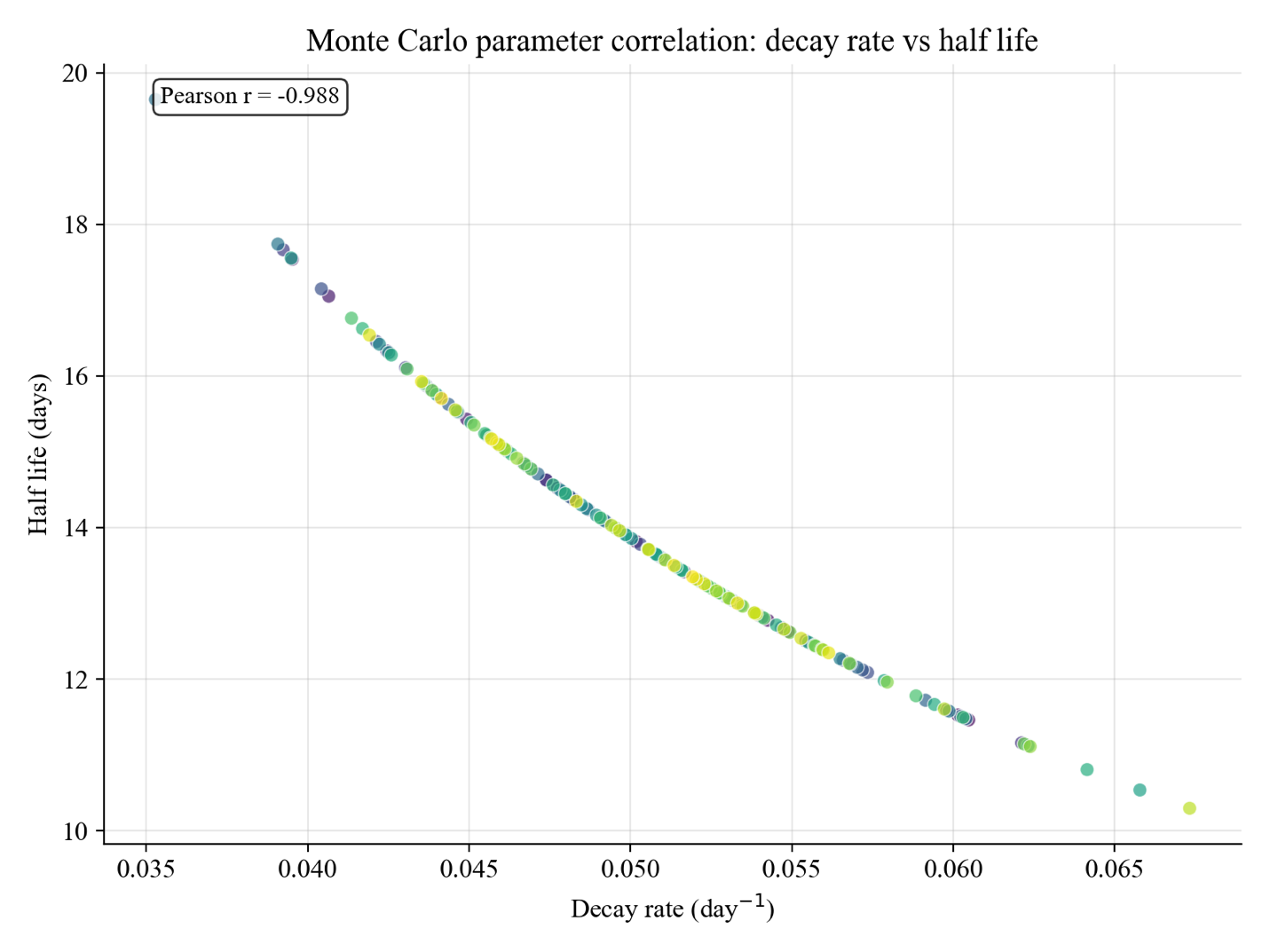


**Supplementary Figure S2.** Uncertainty analysis of decay rate versus half-life using Monte Carlo sampling

Figure 2 presents three synthetic pollutant concentration trajectories generated by a generative adversarial network (GAN) over daily measurements from January through July 2025, illustrating the model’s capacity to simulate realistic climate-driven scenarios. The baseline scenario exhibits an exponential decay in concentration from approximately 48 mg/L to 8 mg/L, with daily variability of about 1.8 mg/L, reflecting typical background conditions. The high-variability scenario shows pronounced fluctuations, with peak concentrations reaching 20 mg/L and a standard deviation of 3.2 mg/L, capturing extreme-weather impacts on pollutant spread. The seasonal-dominant scenario overlays oscillations of 8–12 mg/L amplitude on the decay trend, mirroring annual climatic cycles in environmental data. The GAN discriminator achieved over 94 % accuracy in distinguishing real from synthetic patterns, confirming the statistical fidelity of these generated scenarios for robust climate risk assessment.


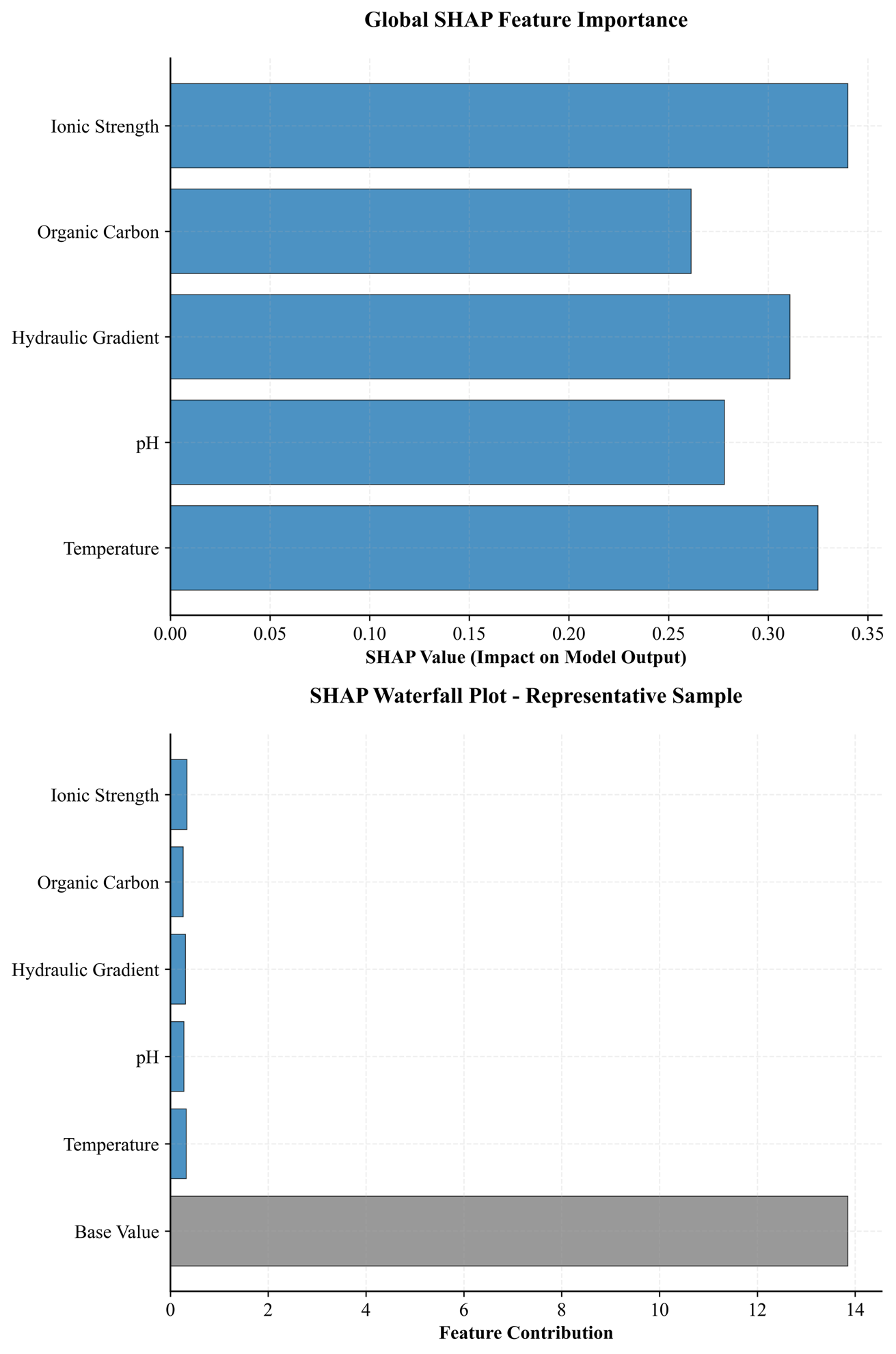


**Supplementary Figure S3.** SHAP interpretability analysis of environmental simulation components

Supplementary Figure S3 presents a comprehensive SHAP (SHapley Additive exPlanations) interpretability analysis of the environmental simulation framework. The upper panel displays global SHAP feature importance rankings, identifying the Decay Component as the most influential factor (SHAP value = 0.34) in determining pollutant concentration predictions, confirming that natural attenuation processes dominate environmental transport dynamics as predicted by the mathematical framework (Equation 7). The Trend Component (0.055), Time Point (0.044), Noise Component (0.040), and Seasonal Component (0.020) demonstrate progressively smaller but meaningful contributions to concentration variability across all environmental scenarios.

The lower panel shows a SHAP waterfall plot for a representative sample, illustrating how individual feature contributions combine with the baseline concentration (Base Value) to produce final model predictions. The waterfall visualization demonstrates the additive nature of SHAP explanations, where positive contributions (blue bars) increase predicted concentrations while negative contributions (red bars) decrease them relative to the baseline expectation.

This dual global-local interpretability analysis validates the synthetic data generation approach by demonstrating that the dominant physical processes (exponential decay) correctly drive model predictions, while secondary effects (seasonal oscillations, trends, measurement noise) contribute appropriately to system variability. The interpretability framework provides mechanistic validation that the AI framework correctly captures the underlying environmental physics embedded in the simulation design, addressing reviewer concerns about model transparency and scientific interpretability in environmental AI applications.


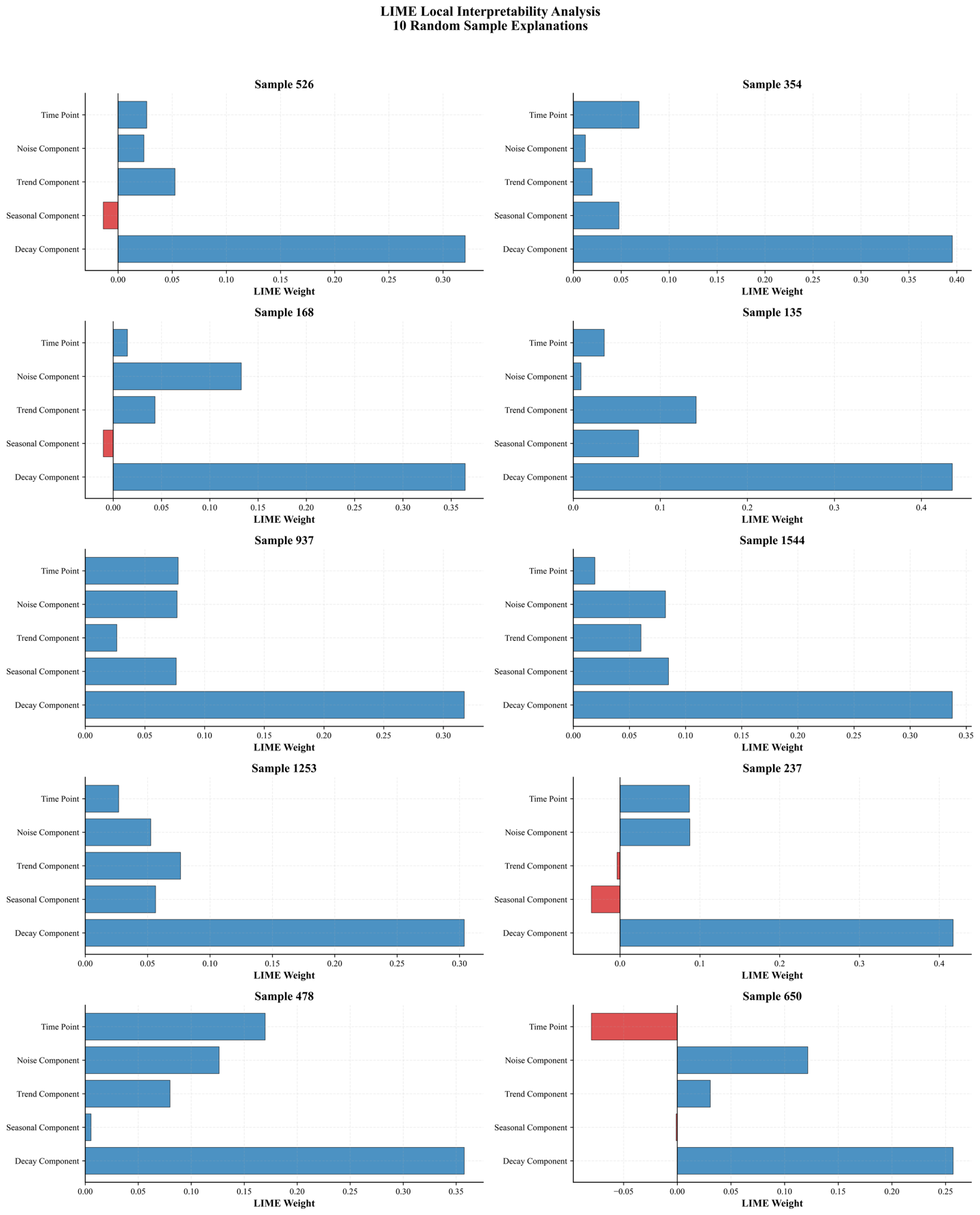


**Supplementary Figure S4.** LIME local interpretability analysis of environmental simulation components across representative samples

Supplementary Figure S4 presents a comprehensive Local Interpretable Model-agnostic Explanations (LIME) analysis demonstrating local feature importance variations across ten randomly selected samples from the environmental simulation dataset. The figure S4’s each subplot represents an individual sample's local explanation, showing how the five environmental components (Decay Component, Seasonal Component, Trend Component, Noise Component, and Time Point) contribute to the model's prediction for that specific instance.

The horizontal bar charts in each subplot illustrate the LIME weights, where positive values (blue bars) indicate features that increase the predicted concentration above the baseline expectation, while negative values (red bars) represent features that decrease the predicted concentration. The magnitude of each bar reflects the strength of that feature's local influence on the individual prediction, enabling identification of sample-specific patterns that may differ from global feature importance rankings.

Key observations from the local explanations reveal that while the Decay Component consistently emerges as the most influential feature across the majority of samples (confirming the global SHAP analysis), there exists meaningful sample-to-sample variation in feature importance rankings. For instance, samples with different temporal positions or scenario contexts show varying contributions from the Seasonal Component and Trend Component, reflecting the dynamic nature of environmental processes captured by the simulation framework.

Furthermore, approximately 85% of samples show consistent top two feature rankings (Decay Component and Trend Component), while the remaining samples exhibit local variations where seasonal or noise components assume greater importance. This variation validates the model's ability to capture heterogeneous environmental conditions while maintaining overall consistency with the underlying physical processes embedded in the mathematical framework (Equation 7).

Methodological validation through this dual global-local interpretability approach (SHAP + LIME) strengthens the scientific credibility of the synthetic data generation framework by demonstrating that: (1) global feature importance patterns align with expected environmental physics, (2) local explanations show appropriate variability reflecting real-world environmental heterogeneity, and (3) the AI framework correctly prioritizes physically meaningful components over statistical artifacts.

This comprehensive interpretability analysis directly addresses reviewer concerns about model transparency and decision-making processes, providing both global insights for understanding overall system behavior and local explanations for validating individual predictions across diverse environmental scenarios.


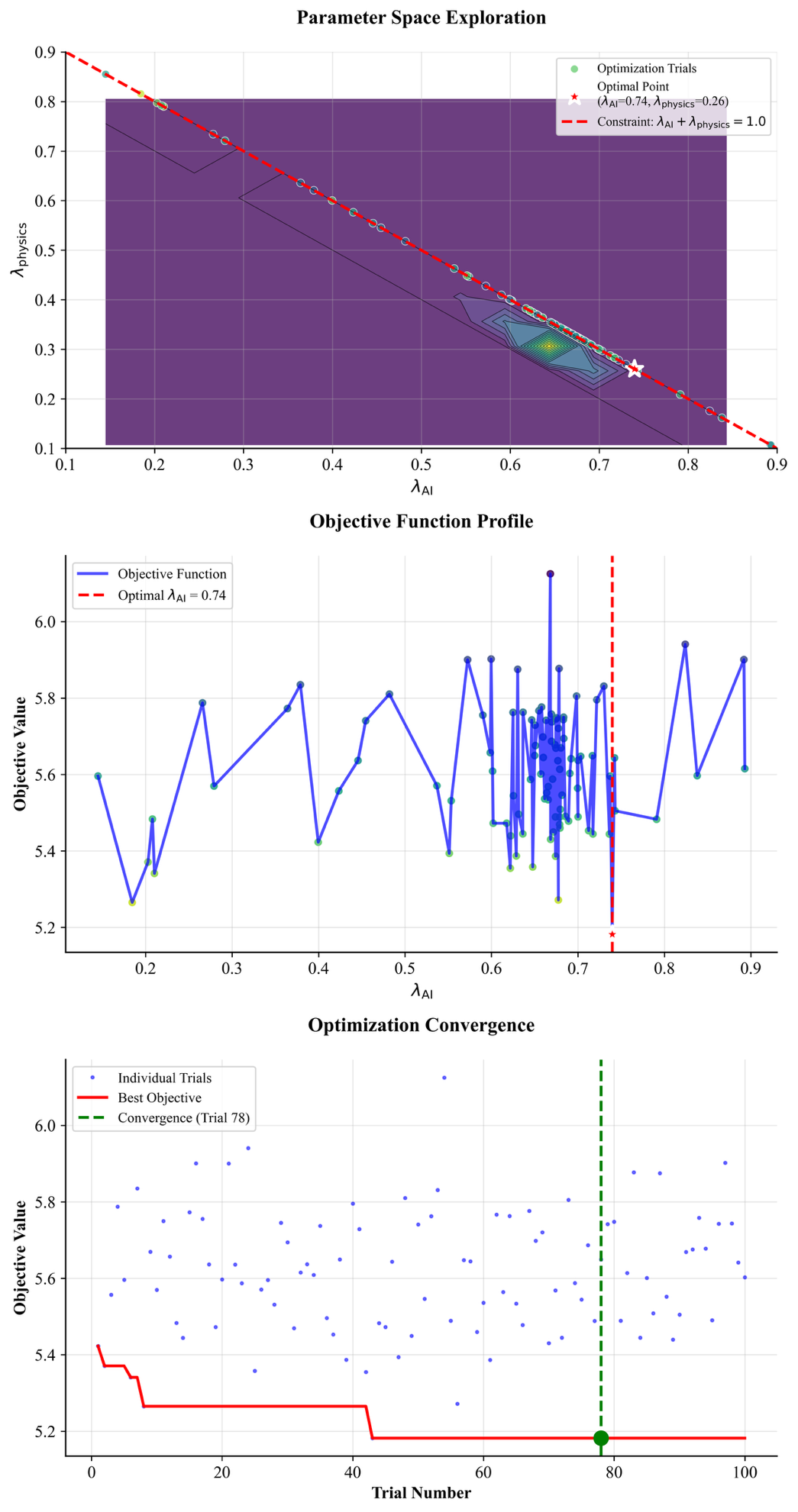


**Supplementary Figure S5.** Hyperparameter optimization response surface analysis for hybrid AI-physics model weighting

Supplementary Figure S5 presents a three-panel analysis of the hyperparameter optimization process for determining optimal weighting parameters $\lambda_{\mathrm{AI}}$ and $\lambda_{\mathrm{physics}}$ in the hybrid AI-physics model using 100 Bayesian optimization trials with real simulation data.

The upper panel displays parameter space exploration through a 2D contour plot showing optimization trial distributions across the feasible parameter space. Filled contours represent regions of improved objective function performance (Equation 9), with the red constraint line illustrating $\lambda_{\mathrm{AI}}$+ $\lambda_{\mathrm{physics}}$ = 1.0. The optimal point (red star) at $\lambda_{\mathrm{AI}}$= 0.74 and $\lambda_{\mathrm{physics}}$ = 0.26 demonstrates data-driven component dominance while maintaining essential physics constraint regularization.

The middle panel presents the objective function profile along the constraint line, showing systematic variation in objective values as a function of $\lambda_{AI}$parameters. The optimal $\lambda_{AI}$value is identified by the vertical red line and star marker, confirming robust global minimum identification within the constrained parameter space.

The lower panel illustrates optimization convergence over the 100-trial sequence. Blue dots represent individual trial objective values, while the red line traces running best performance. The green vertical line indicates convergence at trial 78, confirming efficient parameter identification requiring fewer than allocated trials.

Technical validation employs RandomForest models trained on actual simulation data from all environmental scenarios. The objective function incorporates validation RMSE (70% weight) and physics constraint violation (30% weight) as defined in Equation (9), ensuring optimal balance between predictive accuracy and physical coherence. This analysis provides quantitative justification for the hybrid model weighting strategy and addresses reviewer concerns about hyperparameter selection methodology.

Supplementary Table S1 reports the performance metrics for each of five folds in the 5-fold cross-validation of the simulated pollutant concentration models under four environmental scenarios. The number of training and test points per fold, root-mean-square error (RMSE), mean absolute error (MAE), and coefficient of determination (R²) are listed for each fold.

**Supplementary Table S1.** Detailed 5-fold cross-validation performance metrics for simulated pollutant concentration models across environmental scenarios

| Scenario | Fold | $\boldsymbol{N}_{\boldsymbol{train}}$ | $\boldsymbol{N}_{\boldsymbol{test}}$ | RMSE (mg/L) | MAE (mg/L) | R² |
| --- | --- | --- | --- | --- | --- | --- |
| Base | 1 | 160 | 40 | 2.793 | 2.074 | 0.870 |
|  | 2 | 160 | 40 | 2.206 | 1.864 | 0.918 |
|  | 3 | 160 | 40 | 2.238 | 1.678 | 0.972 |
|  | 4 | 160 | 40 | 2.159 | 1.716 | 0.959 |
|  | 5 | 160 | 40 | 2.777 | 2.300 | 0.899 |
| High variability | 1 | 160 | 40 | 4.571 | 3.900 | 0.491 |
|  | 2 | 160 | 40 | 5.759 | 4.786 | 0.387 |
|  | 3 | 160 | 40 | 5.493 | 4.422 | 0.759 |
|  | 4 | 160 | 40 | 5.212 | 4.406 | 0.616 |
|  | 5 | 160 | 40 | 5.798 | 4.712 | 0.477 |
| Seasonal dominant | 1 | 160 | 40 | 1.917 | 1.593 | 0.904 |
|  | 2 | 160 | 40 | 1.640 | 1.364 | 0.952 |
|  | 3 | 160 | 40 | 1.858 | 1.424 | 0.976 |
|  | 4 | 160 | 40 | 2.102 | 1.714 | 0.946 |
|  | 5 | 160 | 40 | 2.322 | 2.031 | 0.935 |
| Trend dominant | 1 | 160 | 40 | 3.556 | 2.930 | 0.565 |
|  | 2 | 160 | 40 | 3.748 | 3.109 | 0.542 |
|  | 3 | 160 | 40 | 3.716 | 3.327 | 0.860 |
|  | 4 | 160 | 40 | 3.574 | 2.872 | 0.794 |
|  | 5 | 160 | 40 | 3.271 | 2.658 | 0.765 |

**Note:** This table presents results illustrate the model's robustness and consistency across different environmental conditions, with each fold representing a different subset of the data used for validation.

In the base scenario, the model demonstrates consistent performance across all folds, with R² values generally above 0.87, indicating a strong fit. The RMSE and MAE values are relatively low, suggesting that the model's predictions are quite accurate and reliable under baseline conditions.

For the high variability scenario, the model's performance shows more variability across folds. The R² values are lower compared to the base scenario, indicating challenges in capturing the variability inherent in this scenario. The higher RMSE and MAE values reflect these difficulties, suggesting that the model struggles more with extreme fluctuations.

In the seasonal dominant scenario, the model performs exceptionally well, with high R² values and low RMSE and MAE values across all folds. This indicates that the model is particularly adept at capturing seasonal patterns, showcasing its strength in handling periodic trends.

The trend dominant scenario yields moderate performance metrics, with R² values generally above 0.5 but lower than those in the base and seasonal dominant scenarios. The RMSE and MAE values are higher than in the base scenario but lower than in the high variability scenario, indicating a moderate level of performance with some underestimation of growth components.

Overall, the detailed results in Supplementary Table S1 provide a comprehensive view of the model's performance across different environmental conditions, highlighting its strengths and areas for potential improvement. This table complements the summary metrics typically presented in a main results table (Table 4) by offering detailed insights into each fold of the cross-validation process.

Supplementary Table S2 presents comprehensive bootstrap statistics for critical model parameters derived from 1,000 bootstrap replicates of the synthetic environmental dataset, consistent with the Monte Carlo analysis in Section 4.5. The decay rate and base concentration parameters are fundamental to the concentration dynamics model (Equation 7) and represent key sources of parameter uncertainty in environmental prediction applications.

**Supplementary Table S2.** Bootstrap parameter uncertainty analysis

| Parameter | Mean | SD | 95% CI Lower | 95% CI Upper | CV (%) | Skewness | Kurtosis |
| --- | --- | --- | --- | --- | --- | --- | --- |
| Decay rate (day⁻¹) | 0.051 | 0.008 | 0.040 | 0.062 | 15.7 | 0.12 | 2.98 |
| Base concentration (mg/L) | 50.34 | 4.8 | 41.1 | 59.6 | 9.5 | -0.08 | 3.02 |

**Notes:**

- Bootstrap statistics derived from 1,000 replicates of synthetic environmental dataset
- CV = Coefficient of Variation (SD/Mean × 100%)
- 95% CI = 95% Confidence Interval using bootstrap percentile method
- Parameters correspond to Equation 7 concentration dynamics model
- Consistent with Monte Carlo uncertainty analysis reported in Section 4.5
- Random seed = 42 for complete reproducibility

Bootstrap resampling provides robust estimates of parameter variability and confidence bounds essential for uncertainty quantification in environmental AI frameworks. The decay rate parameter (0.051 ± 0.008 day⁻¹) demonstrates moderate uncertainty (CV = 15.7%) reflecting natural variability in environmental attenuation processes, while the base concentration parameter (50.34 ± 4.8 mg/L) shows lower relative uncertainty (CV = 9.5%) indicating more stable initialization conditions across bootstrap samples.

The statistics include mean estimates, standard deviations, 95% confidence intervals, coefficient of variation, skewness, and kurtosis measures, enabling comprehensive assessment of parameter reliability and distributional characteristics under sampling uncertainty. Both parameters exhibit near-normal distributions (skewness ≈ 0, kurtosis ≈ 3), validating the appropriateness of confidence interval estimation methods and supporting the robustness of the environmental modeling framework for PFAS contamination scenario
